# Targeting Nuclear Mechanics Mitigates the Fibroblast Invasiveness in Pathological Dermal Scars Induced by Matrix Stiffening

**DOI:** 10.1002/advs.202308253

**Published:** 2024-02-14

**Authors:** Xiangting Fu, Ali Taghizadeh, Mohsen Taghizadeh, Cheng Ji Li, Nam Kyu Lim, Jung‐Hwan Lee, Hye Sung Kim, Hae‐Won Kim

**Affiliations:** ^1^ Institute of Tissue Regeneration Engineering (ITREN) Dankook University Cheonan 31116 Republic of Korea; ^2^ Department of Nanobiomedical Science and BK21 Global Research Center for Regeneration Medicine Dankook University Cheonan 31116 Republic of Korea; ^3^ Department of Plastic and Reconstructive Surgery Dankook University Hospital (DKUH) Cheonan 31116 Republic of Korea; ^4^ Dankook Physician Scientist Research Center Dankook University Hospital (DKUH) Cheonan 31116 Republic of Korea; ^5^ Department of Biomaterials Science, College of Dentistry Dankook University Cheonan 31116 Republic of Korea; ^6^ Cell & Matter Institute Dankook University Cheonan 31116 Republic of Korea; ^7^ Mechanobiology Dental Medicine Research Center Dankook University Cheonan 31116 Republic of Korea

**Keywords:** cell migration, cellular mechanosensing, matrix stiffness, nuclear lamin, pathological dermal scar

## Abstract

Pathological dermal scars such as keloids present significant clinical challenges lacking effective treatment options. Given the distinctive feature of highly stiffened scar tissues, deciphering how matrix mechanics regulate pathological progression can inform new therapeutic strategies. Here, it is shown that pathological dermal scar keloid fibroblasts display unique metamorphoses to stiffened matrix. Compared to normal fibroblasts, keloid fibroblasts show high sensitivity to stiffness rather than biochemical stimulation, activating cytoskeletal‐to‐nuclear mechanosensing molecules. Notably, keloid fibroblasts on stiff matrices exhibit nuclear softening, concomitant with reduced lamin A/C expression, and disrupted anchoring of lamina‐associated chromatin. This nuclear softening, combined with weak adhesion and high contractility, facilitates the invasive migration of keloid fibroblasts through confining matrices. Inhibiting lamin A/C‐driven nuclear softening, via lamin A/C overexpression or actin disruption, mitigates such invasiveness of keloid fibroblasts. These findings highlight the significance of the nuclear mechanics of keloid fibroblasts in scar pathogenesis and propose lamin A/C as a potential therapeutic target for managing pathological scars.

## Graphical abstract

The stiffened extracellular matrix in keloid tissues mechano‐activates surrounding fibroblasts, particularly altering nuclear mechanics, including a notable decrease in lamin A/C levels. Consequently, this leads to nuclear softening, facilitating the invasive migration of cells through confined environments.

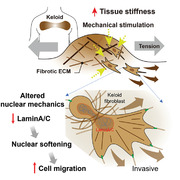

## 1 Introduction

Pathological dermal scars such as keloids exhibit cancer‐like traits, including uncontrolled proliferation, invasiveness, lack of spontaneous regression, and high recurrence rates. They are recognized by abnormally stiffened matrix stemming from thickened, disorganized, and hyalinized collagen bundles.^[^ ^1^, ^2^ ^]^ However, the etiology and molecular mechanisms of keloid formation remain unclear, leading to limited prevention and treatment options.^[^ ^3^ ^]^ Thus far, no single treatments ensure a low recurrence rate; surgical removal alone can have up to a 100% recurrence rate.^[^ ^4^ ^]^ Keloidectomy followed by radiation therapy demonstrates acceptable recurrence rates, yet variability exists among individuals,^[^ ^5^ ^]^ making standardized management challenging. With no effective solutions, keloids cause significant clinical distress; patients often experience itching and pain, and keloid contractures located near the joints lead to severe dysfunction, impacting both physiological and psychological well‐being.^[^ ^6^ ^]^ Skin faces constant external tensions, crucial for homeostasis but also implicated in pathological conditions like scarring.^[^ ^7^ ^]^ Keloids often emerge in mechanically stressed zones, such as the anterior chest, shoulders, upper back, and earlobes,^[^ ^8^ ^]^ where muscle movement, breathing, or earring weight heightens skin tension. Distinct keloid growth patterns thus connect pathogenesis to mechanical force magnitude and direction.^[^ ^8b^ ^]^ Importantly, clinical findings suggest diminishing such forces can notably impede keloid formation and progression.^[^ ^9^ ^]^ For instance, techniques like tension‐reduction suturing curb postsurgical scarring,^[^ ^10^ ^]^ and injecting Botulinum toxin type A into lesions temporarily paralyzes surrounding muscles, reducing tension and preventing keloids.^[^ ^11^ ^]^

Emerging evidence indicates that dysregulated mechanotransduction pathways in keloid fibroblasts (KFs) contribute significantly to keloid formation. Notably, KFs demonstrate elevated YAP/TAZ signaling compared to normal fibroblasts (NFs).^[^ ^12^ ^]^ Moreover, KFs generate increased intracellular force, characterized by enhanced focal adhesion formation and α‐SMA expression.^[^ ^13^ ^]^ The hyper‐responsiveness of KFs to mechanical stimuli, driving keloid progression, also relates to the aberrant expression of the caveolin‐1/ROCK signaling pathway.^[^ ^14^ ^]^ Several studies have thus demonstrated targeting mechanotransduction pathways can alter the pathobiological behaviors of KFs, including hyperproliferation and aggressive migration.^[^ ^13^, ^15^ ^]^ As such, deciphering cellular mechanisms in response to biophysical changes underlying keloid pathogenesis remains an active research area.

Beyond heightened stiffness, keloids are clinically distinct from hypertrophic scars due to their aggressive invasion into surrounding tissues, a cancer‐like trait.^[^ ^16^ ^]^ The invasiveness of KFs is linked to elevated actin filament stiffness and force generation compared to NFs. In vivo migration is particularly challenging amid dense fibrotic extracellular structures.^[^ ^17^ ^]^ Given the intricate microenvironments of keloid tissues, fibroblast migration cannot be solely explained by their contractile traits, rather, cells likely employ multiple mechanisms, responding to matrix properties for migration.

In this study, we investigate the role of matrix stiffness as a biophysical cue in keloid progression, particularly in guiding the confined migration of KFs within keloidal tissues. We find altered nuclear mechanics, specifically lamin‐induced nuclear softening, play a key role in the invasion events of KFs. Such distinct mechano‐pathological traits specific to keloid fibroblasts, particularly in nuclear mechanosensing, explain how these cells aggressively migrate despite the dense ECM of keloidal tissues. Additionally, this insight enables us to identify new potential therapeutic targets for keloid therapy.

## 2 Results

### 2.1 Fibroblasts Are Highly Activated in Keloid Tissues Where the Extracellular Matrix Is Abnormally Stiffened

**Figure 1:**
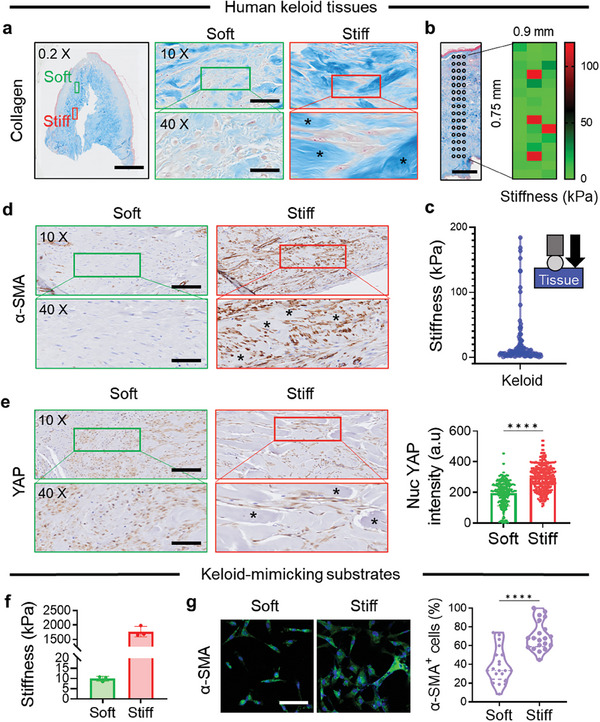
In human keloid tissues, activation of dermal fibroblasts is associated with an abnormal increase in matrix stiffness. a) Masson‘s trichrome staining of human keloid tissues shows heterogeneity in architecture, with regions resembling normal skin tissues and regions with abnormally thick collagen bundles (blue, ^*^). These regions were labeled as “Soft” and “Stiff”, respectively. Scale bar, 2 mm (0.2 ×), 40 µm (10 ×), and 10 µm (40 ×). b,c) Stiffness scanning of human keloid tissues (45 locations with a spacing of 300 µm horizontally and 500 µm vertically in an area of 0.9 mm width × 0.75 mm length) showing a spatial profile of tissue stiffness (b) and heterogeneity in stiffness ranging from 0.5 kPa to 184.2 kPa (*n* = 100 locations) (c). d) Immunohistochemical staining for α‐SMA (brown) in keloid tissue. Scale bar, 40 µm (10 ×), and 10 µm (40 ×). e) YAP staining (left, brown) and quantification of nuclear YAP intensity (right, *n* = 100 cells/condition from 3 independent donors). Scale bar, 40 µm (10 ×), and 10 µm (40 ×). Keloidal thick collagen bundles are indicated by asterisks. f) Stiffness of keloid‐mimicking PDMS substrates (*n* = 3). g) α‐SMA staining (left, green) and quantification (right, *n* = 20 fields/condition from 3 independent experiments) showing increased α‐SMA^+^ cells (%) in keloid fibroblasts cultured on stiff substrates, similar to the in vivo observation. Nuclei are stained with DAPI (blue). Scale bar, 100 µm.^****^ *p* < 0.0001; two‐tailed paired Student's *t*‐test.

Human keloid tissues displayed substantial architectural and stiffness heterogeneity. Normal skin dermis has well‐organized collagen fibers with dense fibroblast populations, resulting in tissue stiffness with a few kilopascals.^[^ ^18^ ^]^ Keloid tissues, in contrast, show an aberrant deposition of thick and densely packed collagen bundles in the dermis (**Figure** **1**a), creating a wide spatial spectrum of tissue stiffness from 0.5 kPa to 184.2 kPa (Figure 1b,c). Regions within keloid tissues mirroring the tissue architecture and stiffness attributes of normal skin tissues are termed “Soft,” whereas those featuring thick collagen bundles and heightened stiffness are designated as “Stiff.” Intriguingly, fibroblasts near thick collagen bundles in stiff regions exhibited higher α‐smooth muscle actin (α‐SMA) expression compared to those in soft regions (Figure 1d; Figure S1a–c, Supporting Information). Furthermore, yes‐associated protein 1 (YAP), a key mechanotransducer sensing matrix stiffness, was more nuclear‐localized in stiff regions (Figure 1e; Figure S1d, Supporting Information), signifying mechano‐activation of fibroblasts due to matrix stiffening.

To investigate the role of matrix stiffness in keloid invasion in vitro, substrates (from 10 kPa to 2 MPa) with covalently conjugated fibronectin were prepared (Figure S2a,b, Supporting Information), ensuring consistent adhesion moiety amounts and densities for cells, regardless of the stiffness (Figure S2c,d, Supporting Information). Both NFs and KFs showed increased adhesion and spreading area as the substrate stiffness increased (Figure S3, Supporting Information). The intrinsic stiffness of keloid tissue samples was measured up to hundreds of kilopascals, while the keloid tissue stiffness can increase up to megapascal scales upon extension.^[^ ^19^ ^]^ Therefore, 10 kPa and 2 MPa were chosen to represent soft and stiff substrates, respectively (Figure 1f). KFs cultured on stiff substrates showed more α‐SMA^+^ cells (Figure 1g), suggesting that KFs respond to matrix stiffness similarly to observations in keloid tissues.

### 2.2 Keloid Fibroblasts Exhibit Higher Mechano‐Sensitivity Compared to Normal Fibroblasts

**Figure 2:**
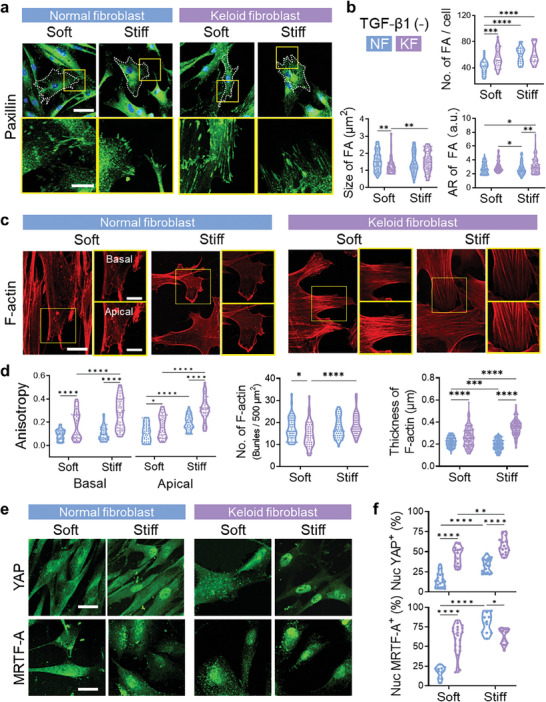
Keloid fibroblasts exhibit elevated mechano‐sensitivity independent of biochemical cues compared to normal fibroblasts. Normal fibroblasts and keloid fibroblasts were stained for paxillin, F‐actin, YAP, and MRTF‐A after 2 days of culture on either soft or stiff substrates in the absence of TGF‐β1. a,b) analysis of focal adhesion (FA) formations. Immunostaining for paxillin (green) (a) and quantification of the number (*n* = 30 FA/cell), size (*n* = 100 FA/condition), and aspect ratio (AR, *n* = 100 FA/condition) of focal adhesions (b). Nuclei are stained with DAPI (blue). Scale bar, 50 µm (main images), and 15 µm (inserts). c,d) Actin development analysis. Representative images of F‐actin staining (red) on the basal and apical planes (c) and quantitative analysis of the F‐actin anisotropy, the number, and thickness of F‐actin bundles (d, *n* = 50–200 cells/condition). Scale bar, 25 µm (main images), and 10 µm (insets). e) Representative images of immunostaining showing intracellular localization of YAP (top, green) or MRTF‐A (bottom, green). Scale bar, 40 µm. f) Quantitative analysis of nuclear YAP or MRTF‐A positive cells (*n* = 10–20 fields/condition). Data are representative of at least three independent experiments. ^*^ *p* < 0.05, ^**^ *p* < 0.01, ^***^ *p* < 0.001, and ^****^ *p* < 0.0001; two‐way ANOVA followed by Tukey's post‐hoc tests.

To examine cellular mechanosensitivity to matrix stiffness in the absence of exogenous biochemical cues, cells were cultured on varying stiffness substrates without transforming growth factor (TGF)‐β1 stimulation. Both NFs and KFs showed increased α‐SMA‐expressing cells on stiff substrates, indicating stiffness‐dependent cellular activation (Figure S4, Supporting Information). Focal adhesion formation and actin development were also enhanced on stiff substrates (**Figure** **2**a–d). Intriguingly, even on soft substrates, KFs formed more focal adhesions, with basal‐to‐apical actin anisotropy surpassing NFs. Moreover, the nuclear translocation of mechanosensitive transcription factors, YAP and myocardin‐related transcription factor A (MRTF‐A), was significantly higher in KFs than in NFs, particularly evident on soft substrates (Figure 2e,f). These findings indicate higher mechanosensitivity of KFs versus NFs. On stiff substrates, both fibroblast types displayed more pronounced mechanoresponses, including focal adhesion formation, actin development, and nuclear translocation of YAP and MRTF‐A. Additionally, nuclear translocation of Smad2/3, a factor linked to the transcription of fibrotic genes, increased in both cell types on stiff substrates (Figure S5, Supporting Information).

Upon TGF‐β1 treatment, the overall level of fibroblast activation was amplified alongside matrix stiffness (Figures S4–S9, Supporting Information). Furthermore, this activation resulted in increased cell proliferation (Figure S10, Supporting Information). KFs exhibited elevated proliferation on stiff substrates regardless of TGF‐β1. However, the proliferation of NFs depended on TGF‐β1; intriguingly, NFs on soft substrates with TGF‐β1 exhibited greater proliferation than those on stiff substrates lacking TGF‐β1 (Figure S10, Supporting Information). These findings suggest NFs rely on TGF‐β1 for activation, while KFs prioritize matrix stiffness over TGF‐β1. Taken all, matrix stiffness serves as a key mechanical cue for KF activation, working synergistically with biochemical cues to regulate cellular behaviors.

### 2.3 Matrix Stiffness Induces Alterations in Nuclear Shape, Followed by Chromatin Reorganization and Histone Modification of Keloid Fibroblasts

**Figure 3:**
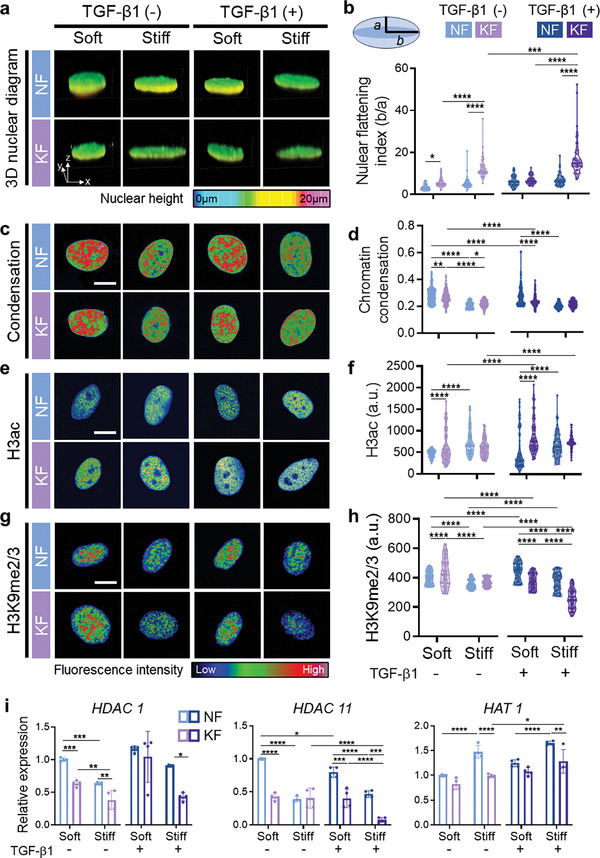
Matrix stiffness triggers changes in the nuclear shape of keloid fibroblasts, which are then accompanied by chromatin reorganization and histone modification. a,b) Nuclear morphology analysis showing color‐coded images of 3D nuclear morphology (a) along with quantitative analysis of nuclear flatting index (b, *n* = 50–56 nuclei/condition). c,d) Representative color‐coded images of DAPI‐stained nuclei (c) subjected to chromatin condensation analysis (d, *n* = 200 nuclei/condition). e–h) Immunostaining for histone 3 acetylation (e, *n* = 200 nuclei/condition) and H3K9 methylation (g, *n* = 100 nuclei/condition), and quantification of mean intensity of the immunostaining (f and h). Scale bar, 10 µm (c, e, and g). i) Gene expression of HDAC 1, 11, and HAT 1 (*n* = 4/condition). Data represent mean ± s.d. of *n* and are representative of at least three independent experiments. ^*^ *p* < 0.05, ^**^ *p* < 0.01, ^***^ *p* < 0.001, and ^****^ *p* < 0.0001; two‐way ANOVA followed by Tukey's post‐hoc tests.

Cells utilize traction forces to perceive the microenvironmental mechanical properties. These forces are transmitted toward the nucleus through cytoskeletal filaments that establish physical connections from the cell periphery to the nuclear envelope.^[^ ^20^ ^]^ Our initial observation revealed matrix stiffness altered the nuclear shape of fibroblasts (**Figure** **3**a,b; Figure S11, Supporting Information). On soft substrates lacking TGF‐β1, both fibroblast types had similar nuclear areas (Figure S11a, Supporting Information). However, KFs displayed smaller nuclear volumes than NFs, indicating more spread and flattened nuclear morphology in KFs (Figure S11b, Supporting Information; Figure 3b), possibly due to increased actin development (Figure 2). Stiff substrates and/or TGF‐β1 treatment further intensified nuclear flattening in both fibroblast types.

Considering that nuclear morphological change can induce epigenetic remodeling and subsequent shifts in cellular behaviors,^[^ ^20^, ^21^ ^]^ we investigated alterations in the epigenetic landscape, such as chromatin condensation and histone modifications (Figure 3c–h). For both cell types, matrix stiffness, rather than TGF‐β1, predominantly influenced chromatin condensation (Figure 3c,d). Regardless of TGF‐β1, fibroblasts on stiff substrates exhibited significant chromatin decondensation with increased histone 3 acetylation (Figure 3e,f), decreased H3K9 and H3K27 methylation (Figure 3g,h; Figure S12, Supporting Information), and elevated H3K4 methylation (Figure S12, Supporting Information), collectively delineating a more open nucleosome structure (euchromatin). Notably, on soft substrates, KFs showed more pronounced euchromatin formation than NFs. This finding suggests that while both fibroblast types exhibited similar trends in epigenetic remodeling, the alterations were more pronounced in KFs compared to NFs. This difference might stem from the fact that KFs generate stronger forces promoting nuclear spreading due to increased actin formation relative to NFs.

Given that matrix stiffness modulates the epigenetic landscape by controlling the expression of histone modifiers,^[^ ^22^ ^]^ we assessed histone deacetylases (HDACs), histone acetyltransferases (HATs), and histone methyltransferases (HMTs) levels (Figure 3i; Figure S13, Supporting Information). For NFs, HDAC1, HDAC11, and HAT1 expression correlated with matrix stiffness (Figure 3i). Specifically, on stiff substrates, NFs exhibited reduced HDAC1 and HDAC11 and increased HAT1, which was unaffected by TGF‐β1 treatment. Conversely, KFs displayed HDAC1 and HDAC11 levels influenced by matrix stiffness and TGF‐β1, while HAT1 remained unaffected by both cues. Notably, KFs consistently showed low HDAC1 and HDAC11 expression across stiffness conditions. HDAC2 and HDAC8 expression did not significantly vary with stiffness but were generally higher in KFs (Figure S13a, Supporting Information). These results imply intrinsic epigenetic distinctions in KFs. Expression of HMTs remained mostly unaffected by matrix stiffness and TGF‐β1 treatment in both fibroblast types (Figure S13b, Supporting Information). Overall, matrix stiffness shapes the epigenetics of dermal fibroblasts primarily through nuclear shape changes, together with chemical cues, influencing HDAC1 and HDAC11 expression synergistically.

### 2.4 Nuclear Shape Alteration of Keloid Fibroblasts is Accompanied by Changes in the Nuclear Lamina and Lamina‐Associated Chromatin Perinuclear Anchoring

**Figure 4:**
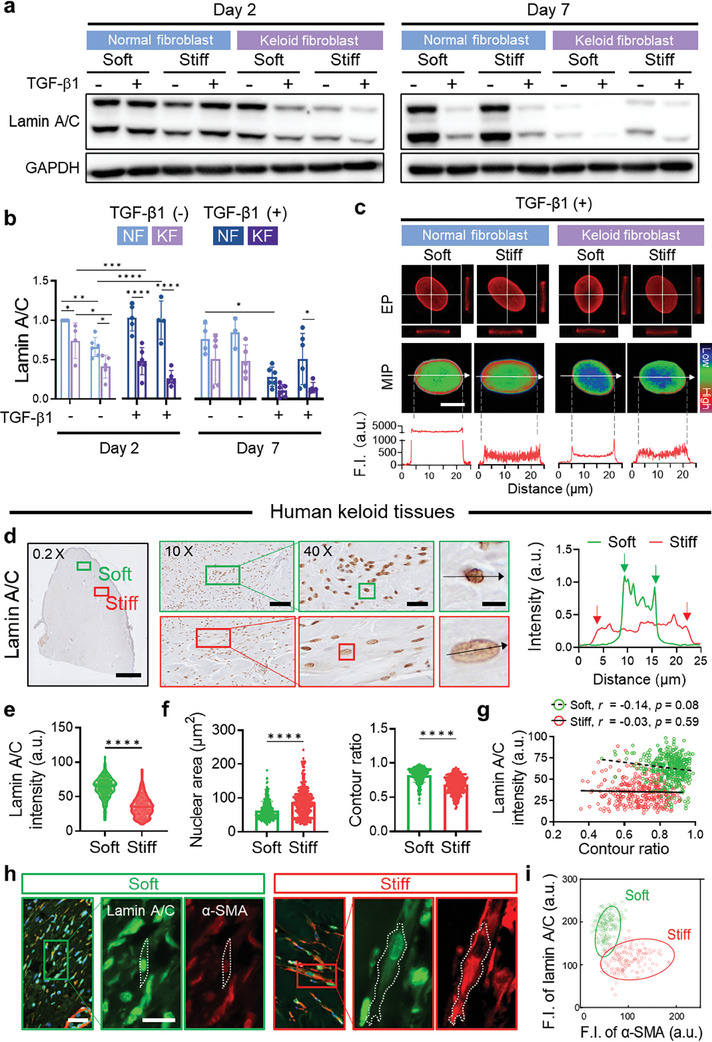
Alteration in the nuclear shape of keloid fibroblasts is associated with modifications in the nuclear lamina. a,b) Lamin A/C expression in NFs and KFs after culture on either soft or stiff substrates for 2 and 7 days. Representative western blots (a) and quantification (b, *n* = 3–6 replicates/condition). c) Representative images of immunostaining for lamin A/C showing its distribution within the nucleus. XZ‐ and YZ‐axes projections on the equatorial plane (EP) of the nucleus (top). Maximum intensity projection (MIP) of a series of z‐stacks images covering the entire nuclear region (middle), along with line scans of fluorescence intensity (F. I.) from the immunostaining images (bottom). Scale bar, 10 µm. Dotted lines mark the edges of the nucleus. d–g) Lamin A/C expression in human keloid tissues. Representative images of immunohistochemical staining for lamin A/C in human keloid tissues (d, left). Scale bar, 2 mm (0.2 ×), 40 µm (10 ×), and 10 µm (40 ×). Line scans showing the intensity profiles of lamin A/C staining in the nucleus (d, right). Arrows mark the edges of the nucleus. The lamin A/C intensity (e), nuclear area (f, left), and contour ratio (f, right) of nuclei quantified based on the immunostaining (*n* = 698 cells/condition from three independent donors). Correlation plots show a negative correlation between lamin A/C intensity and contour ratio in both soft and stiff regions (g). The correlation coefficient (Pearson's *r*) was determined by linear fits as in (g, dash line for Soft, and solid line for Stiff). h) Representative images of co‐immunostaining for lamin A/C (green) and a‐SMA (red) in human keloid tissues. Scale bar, 10 µm (main) and 50 µm (insert). i) Cluster map displaying the correlation of lamin A/C and α‐SMA intensity in soft (green) and stiff (red) regions (*n* = 50–54 cells/condition from three independent donors). Data represent mean ± s.d. of *n* and are representative of at least three independent experiments. ^*^ *p* < 0.05, ^**^ *p* < 0.01, ^***^ *p* < 0.001, and ^****^ *p* < 0.0001; one‐way ANOVA followed by Tukey's post‐hoc tests.

Nuclear flattening, prompted by cytoskeletal actin filaments, was accompanied by changes in the expression and distribution of lamin A/C, key nuclear lamina components. Notably, on day 2 without TGF‐β1, KFs on stiff substrates showed a significant reduction in lamin A/C expression relative to those on soft substrates (**Figure** **4**a,b). TGF‐β1 further lowered lamin A/C expression in KFs on both soft and stiff substrates. Conversely, NFs showed a slight lamin A/C decrease on stiff substrates, with no significant impact from TGF‐β1 on day 2. By day 7, NFs had reduced lamin A/C expression with TGF‐β1 only. NFs on substrates without TGF‐β1 showed consistent lamin A/C expression. However, KFs, regardless of TGF‐β1, displayed significantly reduced lamin A/C expression on both substrates. These findings highlight matrix stiffness primarily regulates lamin A/C expression in KFs, while the expression in NFs is more TGF‐β1‐dependent.

Altered lamin A/C expression caused a remarkable shift in its nuclear distribution. Soft substrates led to lamin A/C accumulation at the nucleus periphery in KFs, whereas, on stiff substrates, it dispersed throughout the nucleoplasm (Figure 4c; Figure S14, Supporting Information). With TGF‐β1 treatment, lamin A/C levels decreased, and its periphery accumulation diminished in KFs on stiff substrates (Figure 4c). In contrast, NFs consistently showed lamin A/C accumulation at the nuclear periphery in all conditions. NFs even with TGF‐β1 on stiff substrates maintained periphery localization. Additionally, a negative correlation between lamin A/C expression and nuclear size is apparent (Figure S15, Supporting Information).

A similar observation was made in human keloid tissues, where fibroblasts near stiff collagen bundles exhibited weaker lamin A/C staining intensity compared to those in softer regions (Figure 4d,e; Figure S16a–c, Supporting Information). Moreover, the nuclear area increased with a reduced contour ratio, indicating larger and more elongated nuclear shapes (Figure 4f). Remarkably, both soft and stiff regions of keloid tissues exhibited a negative correlation between lamin A/C expression and nuclear shape (Figure 4g). This suggests that nuclei with decreased circularity express less lamin A/C. The negative correlation was more pronounced in stiff regions versus soft regions. These results underscore that matrix stiffness plays a crucial role in regulating lamin A/C expression and distribution within KF nuclei.

Particularly in stiff regions of keloid tissues, cells with lower lamin A/C expression also exhibited higher α‐SMA levels compared to those in soft regions (Figure 4h,i; Figure S16d,e, Supporting Information). This suggests that the activation and contractility of KFs are modulated alongside lamin A/C expression during keloid progression. Given lamin A/C's role in anchoring lamina‐associated heterochromatin regions enriched with H3K9me2/3,^[^ ^23^ ^]^ our findings indicate that reduced lamin A/C in KFs on stiff substrates markedly weakened the association between lamin A/C and H3K9me2/3‐marked chromatin (Figure S17, Supporting Information). KFs on stiff substrates with TGF‐β1 displayed substantially reduced H3K9 methylation and disrupted perinuclear anchorage of H3K9me2/3‐marked chromatin, aligned with reduced lamin A/C expression and diminished periphery accumulation. However, KFs overexpressing lamin A/C (LMNA OE) countered the decline in the anchorage of H3K9me2/3‐marked chromatin. In contrast, NFs, with no significant lamin A/C reduction, showed little H3K9 methylation change.

### 2.5 Significant Reduction of Lamin A/C Increases the Nuclear Deformability in Keloid Fibroblasts

**Figure 5:**
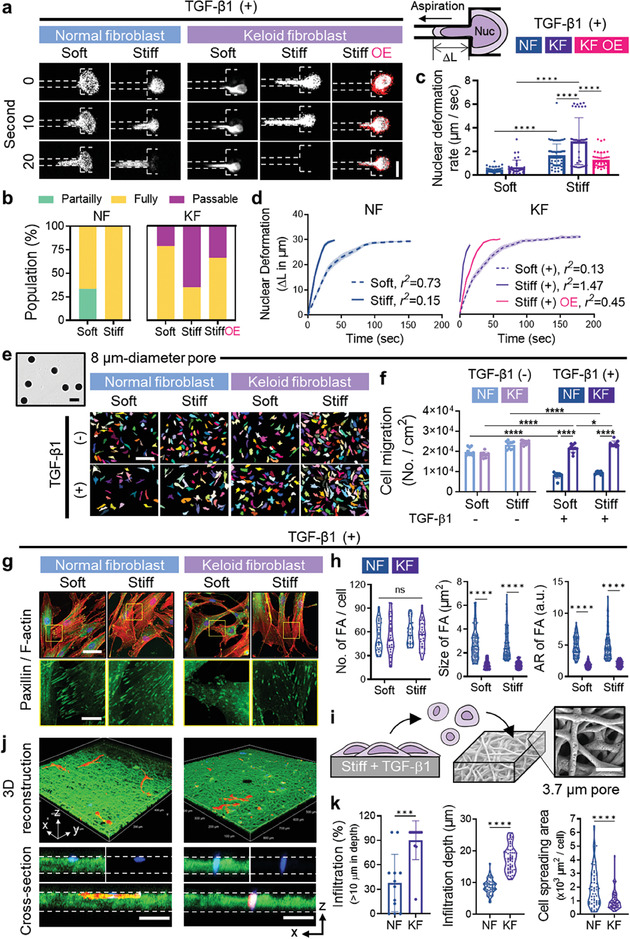
Nuclear softening, along with weak adhesion, accelerates confined migration in keloid fibroblasts. a–d) Nuclear deformability analysis. Representative time‐lapse image series of the deformation of fluorescently labeled cell nuclei in the microfluidic micropipette aspiration device (left) with a schematic (top right) (a). Scale bar, 10 µm. NFs, KFs, or KFs transfected with mCherry‐LMNA plasmid (OE) were pre‐cultured on either soft or stiff substrates for 2 days before the assay. The relative proportions of populations depending on nuclear deformability (b). The nuclear deformation rate was measured based on the protrusion length change over time upon aspiration (c, *n* > 30 nuclei/condition). Nuclear protrusion profiles for 180 sec of aspiration (d, *n* = 22–45 nuclei/condition). The slope (*m*) was determined by linear fits as shown in (d). e,f) Transwell migration assays on cells pre‐cultured under each condition for 2 days. An FE‐SEM image of transwell with (8 µm‐diameter pores) (top left), and pseudocolor images of crystal violet staining (rainbow) following the transwell migration assay (right) (e) and quantification (f, *n* = 10 field/condition). Scale bar, 200 µm. g,h) Analysis of focal adhesion formations in response to TGF‐β1. Representative co‐staining images of paxillin (green) and F‐actin (red) (g). Scale bar, 50 µm (main images), and 15 µm (inserts). Quantitative analysis of the number (*n* = 30 cells/condition), size (*n* = 100 FA/condition), and aspect ratios (*n* = 100 FA/condition) of focal adhesions based on the co‐staining images (h). i–k) 3D confined migration assays through polymeric dense fibrous matrices. Schematic of the assay, along with an FE‐SEM image showing the fibrous structure of the matrix with 3.7 µm‐diameter pores (i). After 2 days of pre‐culture on stiff substrates with TGF‐β1, cells were replated onto the fibrous matrices for the confined migration assay. 3D confocal reconstructions of cell (red) migration through fibrous networks (green) along with cross‐sectional views (j). Nuclei are stained with DAPI (blue). Scale bar, 100 µm. Quantification of the percentage of infiltrated cells (*n* = 11 fields/condition), cell infiltration depth (*n* = 55 cells/condition from 10 independent experiments), and cell spreading area (*n* = 55 cells/condition from 10 independent experiments) (k). Data are representative of at least three independent experiments. ^*^ *p* < 0.05, ^**^ *p* < 0.01, ^***^ *p* < 0.001, and ^****^ *p* < 0.0001; one‐way ANOVA followed by Tukey's post hoc tests for (b), and two‐tailed paired Student's *t*‐test for (f), (h), and (k).

The alterations in chromatin condensation, lamin A/C expression, and perinuclear anchoring of lamina‐associated heterochromatin can potentially impact the nucleus deformability. This was explored using a microfluidic micropipette aspiration assay.^[^ ^24^ ^]^ Cells were pre‐cultured on soft or stiff substrates with or without TGF‐β1 for 2 days before the assay, then detached and aspirated through microchannels (3 µm width, 30 µm length per channel), and the nuclear deformability was assessed by measuring protrusion length (**Figure** **5**a). This assessment specifically measures nuclear deformability without considering adhesion strength.

For NFs pre‐cultured on soft substrates without TGF‐β1, ≈50% had undeformable, 45% partially deformable, and only 5% fully deformable nuclei (Figure 5b; Figure S18, Supporting Information). The proportion of fully deformable nuclei increased with stiffness increase and/or TGF‐β1 treatment. All NFs pre‐cultured on stiff substrates with TGF‐β1 achieved full deformability; however, the nuclear deformation rate for NFs (1.7 µm sec^−1^) was slower than KFs under the same conditions (3.0 µm sec^−1^) (Figure 5c).

Intriguingly, KFs pre‐cultured on soft substrates without TGF‐β1 exhibited 94% fully deformable nuclei – around a 19‐fold increase versus NFs under the same conditions. Furthermore, the remaining 6% of KFs completely traversed the microchannels, showcasing their high nuclear deformability. KF nuclei could be squeezed to pass through the microchannels within 10 s, whereas NF nuclei were deformable but remained stuck within the channels (Figure 5d). Among KFs pre‐cultured on stiff substrates, passable populations were 40% without TGF‐β1 and 70% with TGF‐β1; yet, deformation rates did not significantly differ (2.7 and 3.0 µm sec^−1^ for Stiff (‐) and Stiff (+) in KFs) (Figure S18, Supporting Information). Importantly, overexpressing lamin A/C in KFs prevented the drastic increase in nuclear deformability (Figure 5b–d; Figure S18, Supporting Information). Overall, these findings demonstrate that matrix stiffness reduces lamin A/C expression, leading to increased nuclear deformability in KFs, namely “nuclear softening”.

### 2.6 Nuclear Softening, in Conjunction with Weak Adhesion, Expedites Confined Migration of Keloid Fibroblasts

Based on our findings, we hypothesized that nuclear softening might alter the migration behaviors of KFs, particularly in confined microenvironments where the nucleus experiences physical stress during migration. To validate this, we assessed the migratory ability of KFs through narrow pores using a transwell (8 µm‐diameter pores) migration assay (Figure 5e,f). KFs pre‐cultured on stiff substrates exhibited enhanced confined migration over soft substrates. Upon TGF‐β1 treatment, the KFs pre‐cultured on soft substrates showed increased confined migration, while those on stiff substrates did not respond to additional TGF‐β1 influence. This confirms matrix stiffness alone can activate KFs, particularly confined migration. For NFs, the confined migration also correlated positively with the stiffness of pre‐cultured substrates, but TGF‐β1 treatment significantly reduced the migration, regardless of stiffness, overweighing the stiffness effect.

Immunostaining for paxillin further revealed the distinct focal adhesion patterns in NFs and KFs in response to TGF‐β1 (Figure 5g,h; Figure S19, Supporting Information). Without TGF‐β1, both cell types formed more focal adhesions on stiff substrates, with no notable difference in numbers (Figure S19, Supporting Information). KFs displayed larger, elongated focal adhesion complexes compared to NFs on both soft and stiff substrates. TGF‐β1 treatment did not alter focal adhesion numbers but significantly increased the size and aspect ratio in NFs while decreasing them in KFs (Figure 5g,h. Moreover, matrix stiffness did not induce differences in the focal adhesions in the presence of TGF‐β1. In a scratch‐based migration assay, NFs migrated faster on stiff substrates due to their stiffness‐dependent focal adhesions (Figure S20, Supporting Information), and TGF‐β1 did not accelerate the migration of NFs. However, KFs exhibited faster migration than NFs, regardless of substrate stiffness and TGF‐β1 treatment. In summary, NFs mature focal adhesions in response to TGF‐β1, while KFs reduce them, resulting in diminished migration of NFs and increased mobility of KFs. Coupled with the results of nuclear softening (Figure 4) and increased actin development (Figure 2), the reduced focal adhesion formation in KFs synergistically contributes to increasing their confined migration.

The confined migration of fibroblasts was further challenged by 3D dense fibrous matrices where passageways have 3.7 µm‐diameter pores (Figure 5i–k; Figure S21, Supporting Information). After pre‐culturing on stiff substrates with TGF‐β1 for 2 days, cells were replated onto the fibrous matrices, and migration was monitored for 18 h (Figure 5i). Despite the narrow pore size, KFs could penetrate the matrix to 17.7 µm depth, whereas NFs managed only 8.9 µm (Figure 5j,k). While NFs spread well on the fibrous matrix surface, KFs displayed limited spreading and, instead, progressed substantial nuclear deformation, indicating active 3D confined migration through dense fibrous matrices.

### 2.7 Preventing Lamin A/C‐Driven Nuclear Softening Curbs Aggressive Migration of Keloid Fibroblasts

**Figure 6:**
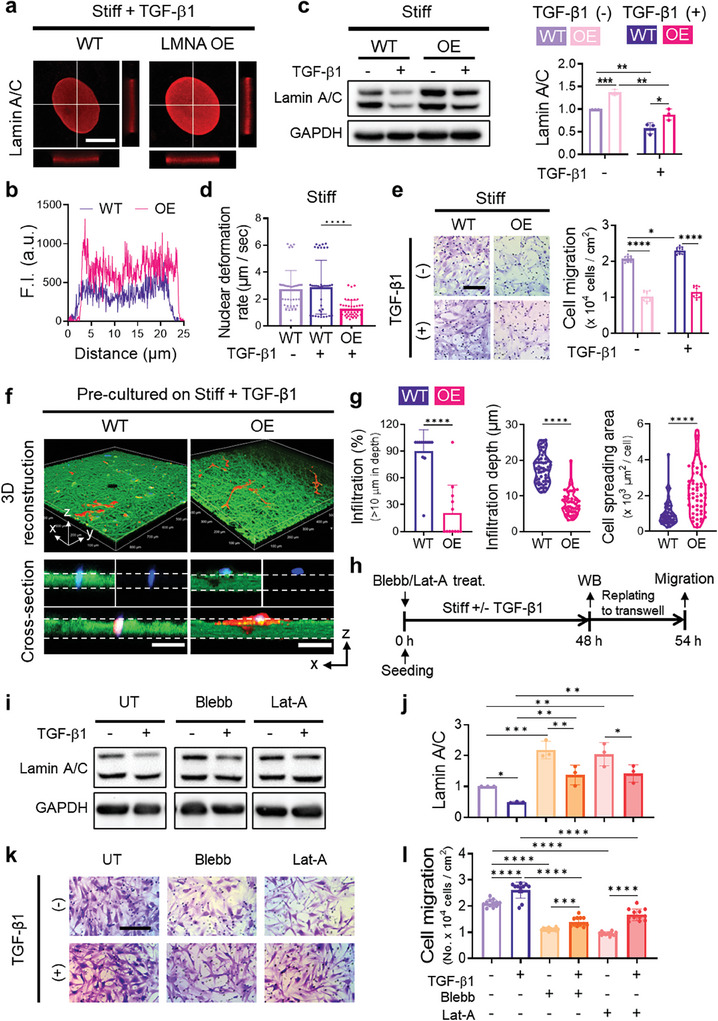
Preventing lamin A/C‐driven nuclear softening blunts the aggressive confined migration behavior of keloid fibroblasts. a) Representative images of immunostaining for lamin A/C (red) in wild type (WT) or lamin A/C‐overexpressed (OE) keloid fibroblasts cultured on stiff substrates in the presence of TGF‐β1 for 2 days. XZ‐ and YZ‐axes projections on the equatorial plane of the nucleus. Scale bar, 10 µm. b) Line scans show the increased lamin A/C intensity and perinuclear accumulation in OE cells compared to WT cells. c) Representative western blots (left) and quantification of lamin A/C expression (right, *n* = 3 replicates/condition). d) Nuclear deformation rates evaluated by the microfluidic micropipette aspiration assay (*n* > 35 cells/condition from 4 independent experiments). e) Representative images of crystal violet staining (purple) following the transwell (8 µm‐diameter pores) migration assay (left) and quantification (right, *n* = 10 fields/condition). Scale bar, 200 µm. f) 3D confocal reconstructions of cell (red) migration through polymeric fibrous networks (green) along with cross‐sectional views. Nuclei are stained with DAPI (blue). Scale bar, 100 µm. g) Quantification of the percentage of infiltrated cells (*n* = 11 fields/condition), cell infiltration depth (*n* = 55 cells from 10 independent experiments), and cell spreading area (*n* = 55 cells from 10 independent experiments). h–l) Indirect regulation of lamin A/C by inhibiting force transmission to the nucleus through the actin cytoskeleton. Timeline showing that blebbistatin (Blebb) or latrunculin A (Lat‐A) were treated to KFs when KFs were seeded on stiff substrates (h). Representative western blots (i) and quantification of lamin A/C in KFs treated with either Blebb or Lat‐A (j, *n* = 3 replicates/condition). Representative images of crystal violet staining (purple) following the transwell (8 µm‐diameter pores) migration assay (k) and quantification (l, *n* = 10 fields/condition). Scale bar, 200 µm. Data represent mean ± s.d. of *n* and are representative of at least three independent experiments. ^*^ *p* < 0.05, ^**^ *p* < 0.01, ^***^ *p* < 0.001, and ^****^ *p* < 0.0001; two‐tailed paired Student's *t*‐test or one‐way ANOVA followed by Tukey's post hoc tests or two‐way ANOVA followed by Tukey's post‐hoc tests.

Considering lamin A/C's pivotal role in nuclear deformability and its influence on confined migration in various human cells, we explored whether restraining lamin A/C‐mediated nuclear softening could curb the aggressive migration of KFs. Initially, we employed LMNA gene overexpression to counteract the reduction of lamin A/C induced by matrix stiffening and/or TGF‐β1 (**Figure** **6**a–c; Figure S22, Supporting Information).

Notably, the nuclear flattening seen in wild‐type KFs due to matrix stiffening or TGF‐β1 was considerably mitigated in LMNA‐overexpressing KFs (Figure S22d, Supporting Information). Moreover, LMNA overexpression decreased nuclear deformability and reinstated H3K9me2/3‐marked chromatin association (Figure 6d; Figure S17, Supporting Information). Consequently, LMNA‐overexpressing KFs exhibited significantly reduced confined migration through transwell (Figure 6e) and dense fibrous matrix (Figure 6f,g). With TGF‐β1, LMNA overexpression did not affect focal adhesions but led to fewer actin fibers while increasing actin thickness compared to the wild type (Figure S22f,g,h, Supporting Information). The results confirm the decisive role of lamin A/C in nuclear deformability and confined migration in KFs.

We also indirectly regulated the lamin A/C levels by disrupting cellular mechanotransduction, specifically force transmission to the nucleus through the actin cytoskeleton (Figure 6h–l). KFs were seeded on stiff substrates simultaneously with the treatment of Latrunculin A (Lat‐A), an actin polymerization inhibitor, or blebbistatin (Blebb), a non‐muscle myosin II ATPase activity inhibitor (Figure 6h). We treated non‐cytotoxic doses of 15 µm Blebb and 40 nm Lat‐A which did not alter KF morphology (Figure S23, Supporting Information). By pharmacologically inhibiting actin polymerization or actomyosin contractility, we prevented lamin A/C reduction in KFs caused by matrix stiffening and/or TGF‐β1 (Figure 6i,j). Consequently, inhibitors‐treated KFs showed markedly reduced confined migration (Figure 6k,l).

We further evaluated potential pharmaceuticals that can either increase lamin A/C expressions or prevent its disassembly (Figure S24, Supporting Information). Mevinolin increases prelamin A/C expression levels, RO‐3306 is a cyclin‐dependent kinase 1 inhibitor, preventing the phosphorylation of lamin A/C, thus limiting its disassembly, and Z‐VEID‐FMK is a caspase‐6 inhibitor, preventing lamin A/C degradation.^[^ ^25^ ^]^ Similar to the effects of LMNA overexpression, the pharmacological inhibition of the reduction in lamin A/C induced by matrix stiffness significantly diminished cell migration. Intriguingly, while both Blebb and Lat‐A also reduced nuclear softening and actomyosin contractility, direct regulation of lamin A/C was more effective in suppressing the confined migration of KFs. These findings imply that lamin A/C‐mediated nuclear softening, triggered by pathological cues like heightened matrix stiffness and TGF‐β1, critically propels KFs to acquire invasiveness within densely packed tissues. Targeting lamin A/C can thus offer a promising clinical approach for keloid therapy.

## 3 Discussion

**Figure 7:**
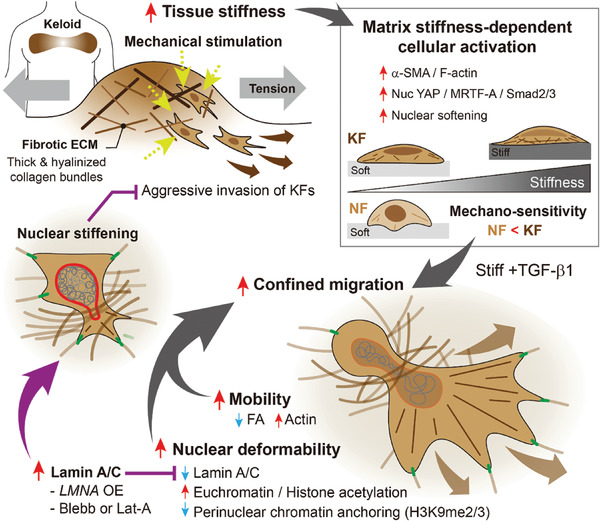
A summary of how matrix stiffness modulates the mechano‐activation and invasive migration of keloid fibroblasts in confining environments. In keloid tissues, keloid fibroblasts (KFs) produce keloidal collagens, which form thickened, disorganized, and hyalinized collagen bundles. These collagens contribute to tissue stiffening. Additionally, keloidal tissues become even stiffer as skin tension intensifies, leading to abnormal mechanical forces exerted on cells. KFs display higher mechanosensitivity to matrix stiffness compared to normal fibroblasts (NFs). Upon TGF‐β1 stimulation, KFs exhibit distinct mechanoresponses, including reduced focal adhesion formation and enhanced actin development. Furthermore, nuclear deformability increases significantly, accompanied by decreased lamin A/C expression, increased euchromatin formation and histone acetylation, and disrupted anchoring of lamina‐associated chromatin. These events collectively facilitate the confined migration of KFs. Importantly, preventing lamin A/C‐driven nuclear softening through LMNA overexpression or actin disruption blunts the invasive migration of KFs.

Living organisms display varying levels of mechanical stress endurance, where relatively stiff tissues like skin, skeletal muscles, and heart bear heightened stress, while more pliable counterparts like the brain remain less resistant.^[^ ^26^ ^]^ Circulating cells, such as immune and cancer cells, encounter fluid shear stress and substantial deformation when navigating confining 3D spaces like constricted cell junctions formed by endothelial cells and connective tissues.^[^ ^17^ ^]^ In fact, cells in their native microenvironment, like dermal fibroblasts, are likely to have evolved distinct mechanisms to sense, adapt, and safeguard their genome against mechanical cues.^[^ ^23^, ^27^ ^]^ The stiffness of keloid tissue mainly results from accumulated thickened, disorganized keloidal collagens synthesized by KFs.^[^ ^1^, ^28^ ^]^ This deposition significantly raises tissue stiffness, particularly under skin tension,^[^ ^19^ ^]^ generating abnormal mechanical forces that impact KFs. These matrix‐mediated mechanics could establish a feedback loop, sustaining activation and invasiveness of KFs in the surrounding skin.^[^ ^29^ ^]^ Our findings illuminate the distinctive mechanoresponsive traits of KFs, marked by heightened sensitivity to matrix stiffness, resulting in enhanced confined migration due to increased nuclear deformability (**Figure** **7**). Thus, this study is considered to bridge the gap in our understanding of the keloid pathogenesis related to markedly altered tissue mechanics, ultimately offering potential therapeutic strategies for keloid treatments.

The nuclear lamina, composed of intermediate filament proteins known as Lamins, forms a structural and mechanical scaffold beneath the nuclear envelope. Given the nuclear lamina is mechanically connected to the extracellular matrix via the cytoskeleton through the LINC complex at the nuclear envelope, the nucleus plays an integral role in sensing and adapting to its extracellular environment.^[^ ^20^, ^30^ ^]^ During deformation, A‐type lamins play a crucial role in nuclear deformability, while B‐type lamins remain relatively constant, maintaining nuclear integrity.^[^ ^31^ ^]^ Thus, A‐type lamins protect the nucleus in mechanically stressed cells. Furthermore, the perinuclear anchoring of chromatin, particularly in regions marked by H3K9me2/3, correlates with lamin A/C levels,^[^ ^23^, ^32^ ^]^ thereby enhancing transient nuclear deformability and protecting against severe deformation induced by external mechanical forces, such as stretching and squeezing.^[^ ^23^ ^]^

During deformation, the nuclear lamina reorganizes, involving the disassembly of lamin A/C at the periphery and a partial detachment of heterochromatin. This reduces heterochromatin marks, increasing nuclear deformability while safeguarding against rupture and DNA damage. Similarly, we observed nuclear softening in KFs due to drastic nuclear shape changes. This is evident from reduced lamin A/C and weakened perinuclear anchoring of H3K9me2/3‐marked heterochromatin under varying matrix stiffness. In KFs, increased histone acetylation drives chromatin decondensation, further contributing to nuclear softening compared to NFs. Remarkably, matrix stiffness and TGF‐β1 collaboratively regulate nuclear morphology and epigenetic traits of KFs.

Nuclear deformability is vital for facilitating cell movement through confining spaces, as the nucleus, being the largest and stiffest organelle, significantly impacts this process.^[^ ^33^ ^]^ Intriguingly, lower lamin A/C levels, common in fluid tissue cell types, contribute to a softer nucleus that enables efficient 3D migration through small pores.^[^ ^34^ ^]^ Recent studies also link reduced lamin A/C levels to tumor metastasis and poor prognosis across various cancer types.^[^ ^35^ ^]^ Similarly, we observed dynamic alterations in lamin A/C expression in KFs, modulating their confined migration responses to matrix stiffness. Our findings suggest that targeting lamin A/C changes triggering nuclear softening might offer a therapeutic strategy to inhibit the invasiveness of KFs. Moreover, a potential avenue exists for diagnosing and prognosticating keloid pathogenesis based on the lamin A/C levels, analogous to the methodologies employed in cancer evaluation.^[^ ^35a^ ^]^

Besides its role in nuclear deformability, A‐type lamin acts as regulatory hubs for transcription factors and signaling pathways.^[^ ^36^ ^]^ Studies have reported that lamin A/C influences actin dynamics through the mechanosensitive serum response factor (SRF) pathway.^[^ ^37^ ^]^ Notably, matrix stiffness, along with myosin‐II activity, modulates lamin A/C phosphorylation and turnover, establishing a feedback loop with actomyosin. In mesenchymal stem cells, lamin A/C and actomyosin engage in a nuclear tension‐dependent manner through the SRF pathway, thereby influencing nuclear and cell morphology in response to matrix mechanics.^[^ ^37b^ ^]^ Our observations align with previous studies that demonstrated changes in F‐actin development based on the expression levels of lamin A/C. Hence, dysregulation and downregulation of lamin A/C could potentially advance keloid progression by disturbing multiple cellular processes in KFs.

Besides nuclear softening, we observed that weak adhesion and high contractility significantly facilitate confined migration of KFs. Physical restriction and low adhesiveness promote a mesenchymal‐to‐amoeboid transition across various cell types, including dermal fibroblasts.^[^ ^38^ ^]^ Without focal adhesions, cortical tension can mechanically delaminate any remaining adhesive sites, leading to a rounded morphology. Additionally, confinement induces a considerable increase in contractility, which in turn accelerates migration. Similarly, weakly adherent cancer cells exhibit adurotactic migration at a faster pace than strongly adherent cells, contributing to enhanced metastatic potential.^[^ ^39^ ^]^ Under pathological conditions, KFs exhibit invasion capacity and plasticity distinct from NFs. The physical attributes of the keloid environment, such as high stiffness and confinement, could trigger a migratory switch in KFs toward a faster and more invasive mode of migration.

The notable nuclear softening and frequent/severe nuclear deformation during the invasion process raise the risk of nuclear envelope rupture and DNA damage.^[^ ^40^ ^]^ This sequence could further exacerbate genetic instability and anomalies within keloids. Previous studies suggest that altering nuclear mechanics through chromatin organization adjustment holds promise for regulating confined cell migration behaviors.^[^ ^41^ ^]^ However, this approach not only impacts nuclear stiffness but also induces substantial alterations in global gene expression profile and epigenetic features. This implies a possibility of unintended consequences, potentially exacerbating genetic instability in keloid fibroblasts. While the influence of genetic and epigenetic backgrounds of individuals on the mechanoresponses of KFs remains open for future studies, our findings provide insights into how KFs’ distinctive mechanoresponses align with their aggressive invasion through confining microenvironments in keloid progression.

Unfortunately, the absence of standardized animal models, attributed to the human‐specific nature of this disease, constrained our ability to conduct in vivo experiments.^[^ ^42^ ^]^ In future studies, the application of in vitro platforms designed to mimic keloid pathogenesis through biomaterials and biotechnology may be an alternative approach to circumvent this challenge.^[^ ^43^ ^]^ The newly identified mechanobiological mechanisms underlying the pathogenesis of dermal scar fibroblasts emphasize the significance of nuclear mechanics, particularly alterations in lamin A/C, which can thus be regarded as a potential therapeutic target for the management of pathological cutaneous scarring (Figure 7). Furthermore, beyond keloids, the conceptual and mechanistic views uncovered herein promise to broaden our understanding of mechano‐pathological cascades in diverse scarring diseases.

## 4 Experimental Section

### Human Keloid Biopsy Analysis

Earlobe keloid biopsies were obtained by the approved protocols of the institutional review board at Dankook University Hospital (IRB No. DKUH 2022‐09‐015) with informed consent from all participants (See Table S1, Supporting Information, for donor information). Human keloid biopsies were fixed in 10% (v/v) neutral buffered formalin, dehydrated in a series of graded ethanol, and then embedded in paraffin. Paraffinized tissues were sectioned with a thickness of 5 µm using a microtome (Leica RM2245, Leica Biosystems, Germany) and stained with Masson's trichrome (Polyscience). For immunostaining of α‐SMA, YAP, and lamin A/C, sectioned slides were incubated with primary antibodies diluted in 1% (w/v) bovine serum albumin (BSA) and incubated for 16 h at 4 °C (See Table S2, Supporting Information, for detailed information on primary antibodies). After subsequent incubation with an HRP‐conjugated anti‐rabbit secondary antibody (Agilent) for 1 h at room temperature, the slide was developed with liquid DAB+ (Agilent) and counter‐stained with hematoxylin. Slides were imaged using a slide scanner (VS200, Olympus, Japan). The mean intensity of YAP and lamin A/C in the nucleus was determined by Fiji after color deconvolution using QuPath (Centre for Cancer Research & Cell Biology at Queen's University Belfast, UK). To measure nuclear circularity, the nuclear area and nuclear perimeter were selected by the freehand selection tool and measured by particle analysis through Fiji, and the contour ratios were calculated as shown in the equation: Contour ratio = 4 π x nuclear area / nuclear perimeter^2^.

For tissue stiffness measurement, fresh biopsies were treated in a 30% (w/v) sucrose solution for 2 weeks, cryosectioned to 20 µm thick slices, and fixed on a coverslip. The tissue sample was immersed in PBS in a 6‐well plate, and the stiffness was measured using a mechanical screening platform (Pavone, Optics11 Life, Netherlands) with a probe (3.92 N m^−1^ stiffness and 27 µm tip radius). For stiffness scanning, a total of 45 locations were scanned with a spacing of 500 µm vertically and 300 µm horizontally in an area of 0.9 mm length × 0.75 mm width. The average stiffness of tissues was determined based on the data from 100 randomly selected locations for the measurement.

### Preparation and Characterization of PDMS Substrates

PDMS substrates with stiffness ranging from 10 kPa to 2 MPa were prepared by blending Sylgard 184 and Sylgard 527 silicone elastomer kits (Dow Corning) as described in Figure S2 (Supporting Information). After adding the curing agent, the mixture was thoroughly mixed, degassed, and cured for 72 h at room temperature. For surface functionalization with fibronectin (FN), the solidified PDMS substrates were treated with oxygen plasma for 5 min using a plasma system (CUTE, Femto‐Science, Inc., South Korea) and incubated with 10% (v/v) (3‐aminopropyl) triethoxysilane (Sigma–Aldrich) for 3 h at 50 °C. The substrates were then incubated in 2.5% (v/v) glutaraldehyde solution (Sigma–Aldrich) for 1 h at room temperature and subsequently treated with 10 µg mL^−1^ FN solution (Thermo Fisher) for 12 h at 4 °C. Hydrophilicity of the surface of FN‐functionalized substrates was evaluated by measuring water contact angles (PHX300, South Korea). HiLyte Fluor 488‐labeled FN (Cytoskeleton) was used to characterize the distributions and concentrations of FN modified on the substrates. The FN density was calculated through the difference in fluorescence intensities (*λ*_em_ = 500 nm) of the supernatant before and after the functionalization.

### Fibroblasts Culture

Primary human dermal fibroblasts isolated from healthy skin or keloids were purchased from Fisher Scientific or ATCC, respectively (See Table S3, Supporting Information, for detailed information on cells). Normal dermal fibroblasts (NFs) were cultured in Dulbecco's Modified Eagle Medium with high glucose (DMEM‐HG, Gibco) containing 10% (v/v) fetal bovine serum (FBS, Corning), 1% (v/v) Penicillin/Streptomycin (P/S, Gibco), 1% (v/v) MEM non‐essential amino acids solution (Gibco), 2 mm Glutamax (Gibco), and 0.1 mm 2‐mercaptoethanol (Gibco). Keloid fibroblasts (KFs) were cultured in DMEM‐HG containing 15% (v/v) FBS and 1% (v/v) P/S. Cells were incubated at 37 °C in a humidified atmosphere of 5% CO_2_, and the medium was changed every 2–3 days. Cells were seeded onto the FN‐functionalized substrates at a density of 1 × 104 cells cm^−2^ for all experiments. If needed, TGF‐β1 (Peprotech) was supplemented in the media at a concentration of 10 ng mL^−1^. After culture for 1, 2, or 4 days, the cell proliferation was assessed using a cell counting kit‐8 (CCK‐8, Dojindo Molecular Technologies, Inc.).

### Immunofluorescence Staining

After 2 days of culture on the substrates, cells were fixed in 4% (v/v) paraformaldehyde for 15 min at room temperature, rinsed with PBS, and then permeabilized with 0.1% (v/v) TritonX‐100 in PBS for 8 min. After washing with PBS, cells were blocked with 1% (w/v) BSA for 45 min at room temperature. After cells were incubated with primary antibodies for 16 h at 4 °C, they were incubated with secondary antibodies for 1 h at room temperature (See Table S4, Supporting Information, for details of antibodies). Subsequently, F‐actin and/or nuclei were stained with Alexa Fluor 546 Phalloidin (A22283, Invitrogen) and 4′,6‐Diamidino‐2‐Phenylindole, Dihydrochloride (DAPI, Thermo Fisher), respectively. Fluorescence images were obtained by fluorescence microscopy (IX73, Olympus, Japan) or confocal laser scanning microscopy (CLSM) (Eclipse Ti2, Nikon, Japan).

### Image Analysis

Image analysis was performed using Fiji (Image J). The number of cell adhesion and cell spreading area was determined based on the F‐actin‐stained cells with the multi‐point tool and freehand selection tool. Focal adhesion formation was analyzed by measuring the number, size, and aspect ratio of paxillin staining with the multi‐point tool, freehand selection tool, and particle analysis. Anisotropy of F‐actin on the basal and apical planes was quantified based on z‐stacks (2.23 µm steps) of F‐actin‐stained images with the plugin FibrilTool. The number and thickness of F‐actin bundles were measured using the multi‐point tool and straight‐line selection tool, respectively. Cells with nuclear localization of YAP, MRTF‐A, and Smad2/3 were counted with the multi‐point tool, and the percentage was calculated as shown in the equation: Nuc YAP/MRTF‐A/Smad2/3^+^ (%) = number of cells with nuclear staining / total number of cells × 100%.

Nuclear morphology was analyzed by measuring nuclear area, volume, and flattening index. DAPI‐stained nuclei were captured with z‐stacks (0.3 µm steps) by CLSM, and the nuclear area, volume, nuclear height (H), and axis (L) were measured by NIS‐Elements software 5.3 (Nikon, Japan), and the flattening index was calculated as shown in the equation: Flattening index = H/L. Chromatin condensation was analyzed by dividing the mean intensity by the standard deviation of DAPI‐stained nuclei. Histone acetylation (H3ac) and methylation (H3K9me2/3, H3K4me3, and H3K27me3) were evaluated by measuring the mean intensity of the immunostaining.

### Quantitative Reverse Transcription Polymerase Chain Reaction (qRT‐PCR)

Total RNA was isolated using Direct‐zol RNA Microprep Kits (Zymo Research), and the cDNA was synthesized using Accupower RT premix (Bioneer) or iScript cDNA Synthesis Kit (Bio‐Rad) according to the manufacturer's protocol. qRT‐PCR was performed with SensiMixTM SYBR Hi‐ROX kit (QT‐605‐05, Bioline) by StepOne Plus real‐time PCR system (Applied Biosystems). Fold changes in gene expression were calculated using the 2^–ΔΔCt^ method with normalization to GAPDH. The primers used are described in Table S5 (Supporting Information).

### Evaluation of Lamin A/C Expression and Distribution

For western blotting, cells were lysed on ice in RIPA lysis buffer (ELPIS biotech) containing Halt Protease and Phosphatase Inhibitor Cocktail (Thermo Scientific). After the lysate was centrifuged at 1 00 000 rpm for 10 min, the protein concentration of the supernatant was quantified using a Pierce BCA protein assay kit (Thermo Scientific). After denaturation by heating for 10 min at 105 °C, an equal amount of total proteins per lane was electrophoresed in polyacrylamide gels and transferred onto nitrocellulose membranes. The membranes were blocked with 5% (w/v) BSA (SolMate BSA fraction IY, GeneAll) prepared in 1x Tris‐Buffered Saline containing 0.1% Tween (TBST) for 45 min, and then incubated with primary antibodies specific for lamin A/C (1: 1000, ab108595, Abcam) and GAPDH (1:1000, sc‐25778, Santa Cruz) in a blocking buffer for 12 h at 4 °C. An anti‐rabbit IgG, HRP‐linked secondary antibody (7074, Cell signaling) diluted in 1x TBST was subsequently treated and incubated at room temperature for 1 h. After washing, protein signals were visualized using Supersignal west pico plus chemiluminescent substrate (Thermo Scientific) and captured by the iBright 1500 imaging system (Thermo Fisher). Densitometry analysis of the western blots was carried out with iBrightTM Analysis Software 4.0 (Thermo Fisher). The lamin A/C distribution was observed by scanning the intensity profiles of lamin A/C immunostaining images with NIS‐Elements software 5.3 (Nikon, Japan). The frequency of lamin A/C staining intensity and co‐localization of lamin A/C with H3K9me2/3 were measured by Fiji.

### Microfluidic Micropipette Aspiration Assay

For the assay, the microfluidic micropipette aspiration device was prepared as previously reported.^[^ ^24^ ^]^ Cells were pre‐cultured on substrates for 2 days prior to the assay. Nuclei were stained with Hoechst 33342 (1:10 000, Thermo Fisher) for 20 min, and cells were then trypsinized and dissociated into single cells. After plasma treatment to the device for 5 min, cell suspension (2 × 106 cells mL^−1^, 12.5 μL min^−1^) was immediately flown with perfusion buffer (6.5 μL min^−1^) through the device. Time‐lapse images were taken every 5 s by CLSM, and the nuclear deformation rate was calculated by measuring the length of protrusion upon aspiration over time through Fiji.

### Migration Assays

For the scratch‐based planar migration assay, cells were plated on the substrates fabricated in a 12‐well plate at a density of 1 × 105 cells per well. After 2 days of culture, the confluent monolayer was scratched with a p200 pipette tip, and the migration was imaged using JuLI Stage (NanoEnTek lnc.) every 2 h for a total of 24 h.

For the transwell migration assay, cells were pre‐cultured on the substrates for 2 days, trypsinized, and re‐plated into an insert well (8 µm‐diameter pores, Corning) at a density of 8 × 104 cells per well. After 6 h of migration, cells were fixed, stained with 6 mM crystal violet (C0775, Sigma–Aldrich) for 20 min at room temperature, and imaged using an inverted microscope (IX73, Olympus, Japan).

For the 3D confined migration assays, fibrous matrices were fabricated by electrospinning polycaprolactone (PCL). Briefly, PCL (Mn 80000, Sigma–Aldrich) was dissolved in a mixture of chloroform and dimethyl sulfoxide (9:1, v/v) at a concentration of 12% (w/v), and then electrospun at a flow rate of 1.5 mL h^−1^ under 13 kV. PCL fibers were collected on a stationary plate and completely dried before use. Before cell seeding, the fibrous matrices were treated with oxygen plasma for 10 min and subsequently incubated in a 20 µg mL^−1^ fibronectin solution for 12 h at 4 °C. Cells pre‐cultured on the substrates for 2 days were trypsinized and re‐plated onto the fibrous matrices at a density of 2800 cells cm^−2^. After 18 h of migration, cells were stained with Alexa Fluor 546 Phalloidin and DAPI and imaged by CLSM. The infiltration percentage and depth were measured by NIS‐Elements software 5.3 (Nikon, Japan). The cell spreading area and aspect ratio were quantified by the Freehand Selection Tool and particle analysis of Fiji.

### Lamin A/C Plasmid Transfection

For transfection, KFs were seeded on PDMS substrates in a 24‐well plate at a density of 2 × 104 cells per well. After 12 h of culture, mCherry LaminA‐C‐18 plasmid (55068, AddGene) was transfected using lipofectamine 3000 (L3000001, Invitrogen) at a concentration of 0.8 µg per well. After 36 h, the transfection was evaluated by fluorescence microscopy and western blotting. All in vitro experiments were performed 36 h after transfection.

### Inhibitor Treatments

Blebbistatin (Blebb, 1760, TOCRIS), Latrunculin A (Lat‐A, 3973, TOCRIS), Mevinolin (M2147, Sigma–Aldrich), RO‐3306 (AG‐CR1‐3515, AdipoGen), Z‐VEID‐FMK (FMK006, Bio‐Techne) were used as potential pharmaceuticals. For dose screening, KFs were seeded on a 96‐well plate at a density of 3000 cells per well and treated with Blebb (2.5, 5, 10, and 15 µm), Lat‐A (10, 20, 30, and 40 nm), Mevinolin (0.5, 1, 2, and 2.5 µm), RO‐3306 (5, 10, 20, and 25 µm), or Z‐VEID‐FMK (1.25, 2.5, 5, and 10 µm). After 48 h of treatment, cell viability was evaluated using the CCK‐8 assay. When KFs were seeded onto stiff substrates, 15 µm Blebb, 40 nm Lat‐A, 2 µM Mevinolin, 5 µm RO‐3306, or 10 µm Z‐VEID‐FMK was simultaneously supplemented in the media at cell seeding. After 2 days of treatment, western blotting and transwell migration assays were performed to evaluate the inhibitory effect.

### Statistical Analysis and Illustrations

All values were expressed as the mean ± standard deviation unless otherwise indicated. Statistical evaluations of the data were performed using two‐tailed Student's *t*‐test, one‐way or two‐way ANOVA followed by Tukey's post‐hoc, Dunnett, or Bonferroni test (GraphPad Prism). In all cases, *p* < 0.05 was considered statistically significant.

## Conflict of Interest

The authors declare no conflict of interest.

## Supporting information

Supporting Information

## Data Availability

The data that support the findings of this study are available from the corresponding author upon reasonable request.
