# Supplementary material for: Targeting Nuclear Mechanics Mitigates the Fibroblast Invasiveness in Pathological Dermal Scars Induced by Matrix Stiffening
Source: Adv Sci (Weinh). 2024 Feb 14;11(15):2308253. doi: 10.1002/advs.202308253 (PMC11022731; doi:10.1002/advs.202308253)
Supplement: Supplementary file 1 — Supporting Information [file ADVS-11-2308253-s001.pdf]

ADVANCED SCIENCE
Open Access

Supporting Information

for *Adv. Sci.*, DOI 10.1002/advs.202308253

Targeting Nuclear Mechanics Mitigates the Fibroblast Invasiveness in Pathological Dermal Scars Induced by Matrix Stiffening

Xiangting Fu, Ali Taghizadeh, Mohsen Taghizadeh, Cheng Ji Li, Nam Kyu Lim, Jung-Hwan Lee, Hye Sung Kim and Hae-Won Kim**

Supporting Information (Figs. S1-S24 &Table S1-S5)

Targeting nuclear mechanics mitigates the fibroblast invasiveness in pathological dermal scars induced by matrix stiffening

Xiangting Fu[1,2,3], Ali Taghizadeh[1,2,3], Mohsen Taghizadeh[1,2,3], Cheng Ji Li[1,2,3], Nam Kyu Lim[4,5], Jung-Hwan Lee[1,2,3], Hye Sung Kim[1,2,3,5,*], Hae-Won Kim[1,2,3,5,*]

[1]Institute of Tissue Regeneration Engineering (ITREN) | [2]Mechanobiology Dental Medicine Research Center | [3]Department of Nanobiomedical Science and BK21 Global Research Center for Regeneration Medicine, Dankook University, Cheonan 31116, Republic of Korea, [4]Department of Plastic and Reconstructive Surgery | [5]Dankook Physician Scientist Research Center, Dankook University Hospital (DKUH), Cheonan 31116, Republic of Korea

----

*Correspondence: hskim1213@dankook.ac.kr (H. S. Kim) and kimhw@dku.edu (H-.W. Kim)

Supporting Information (Figs. S1-S24 &Table S1-S5)

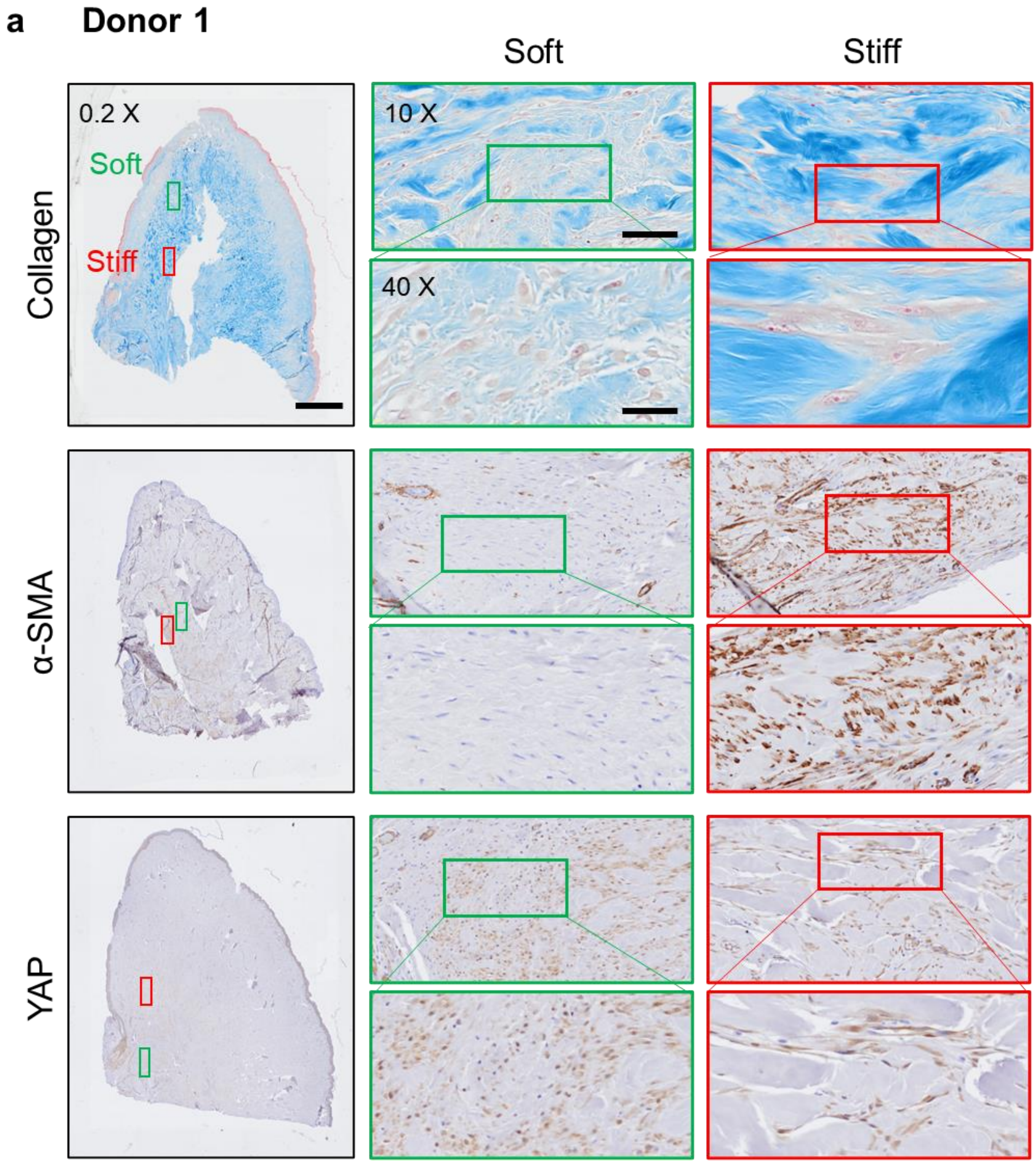

Supporting Information (Figs. S1-S24 &Table S1-S5)

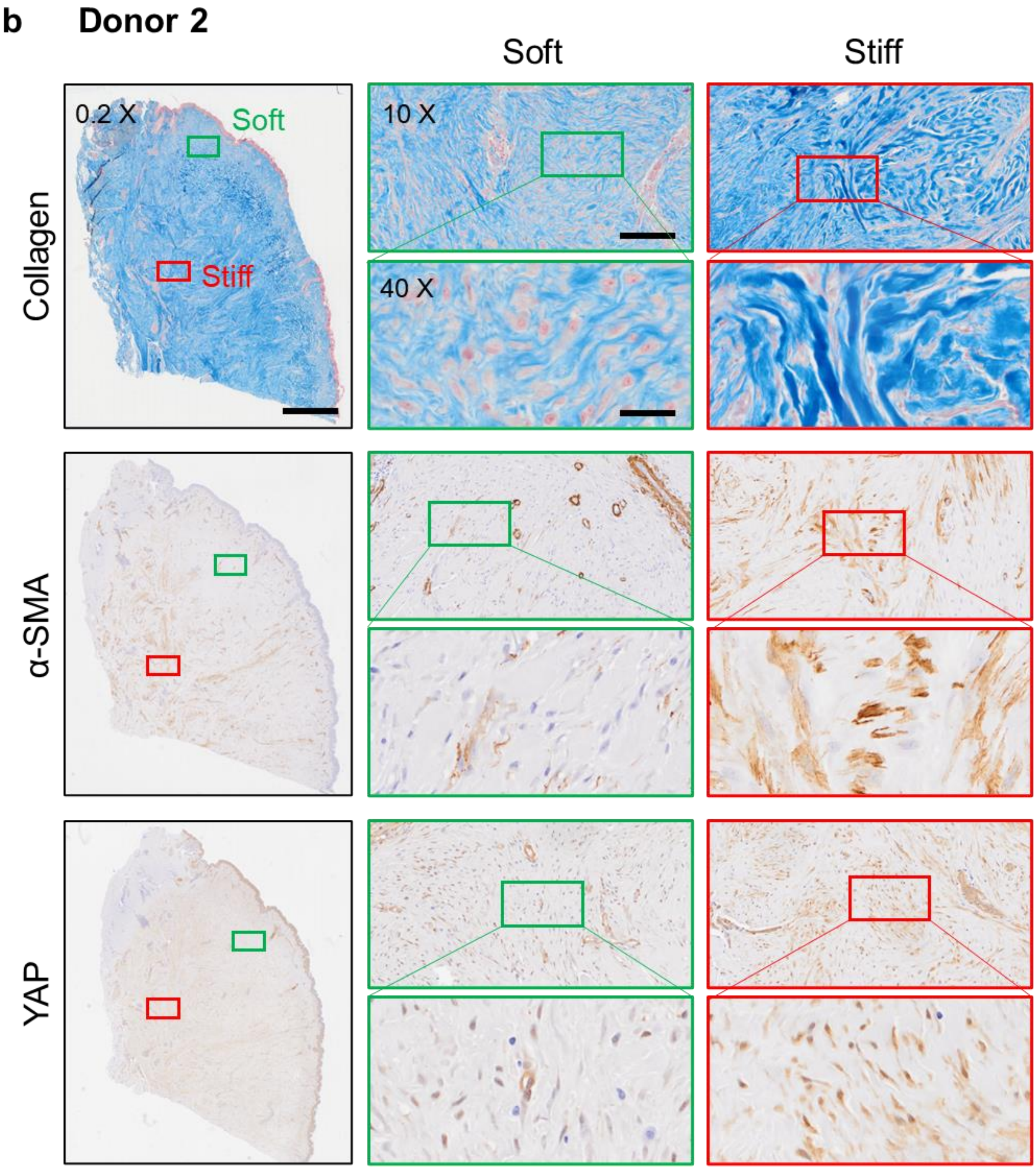

Supporting Information (Figs. S1-S24 &Table S1-S5)

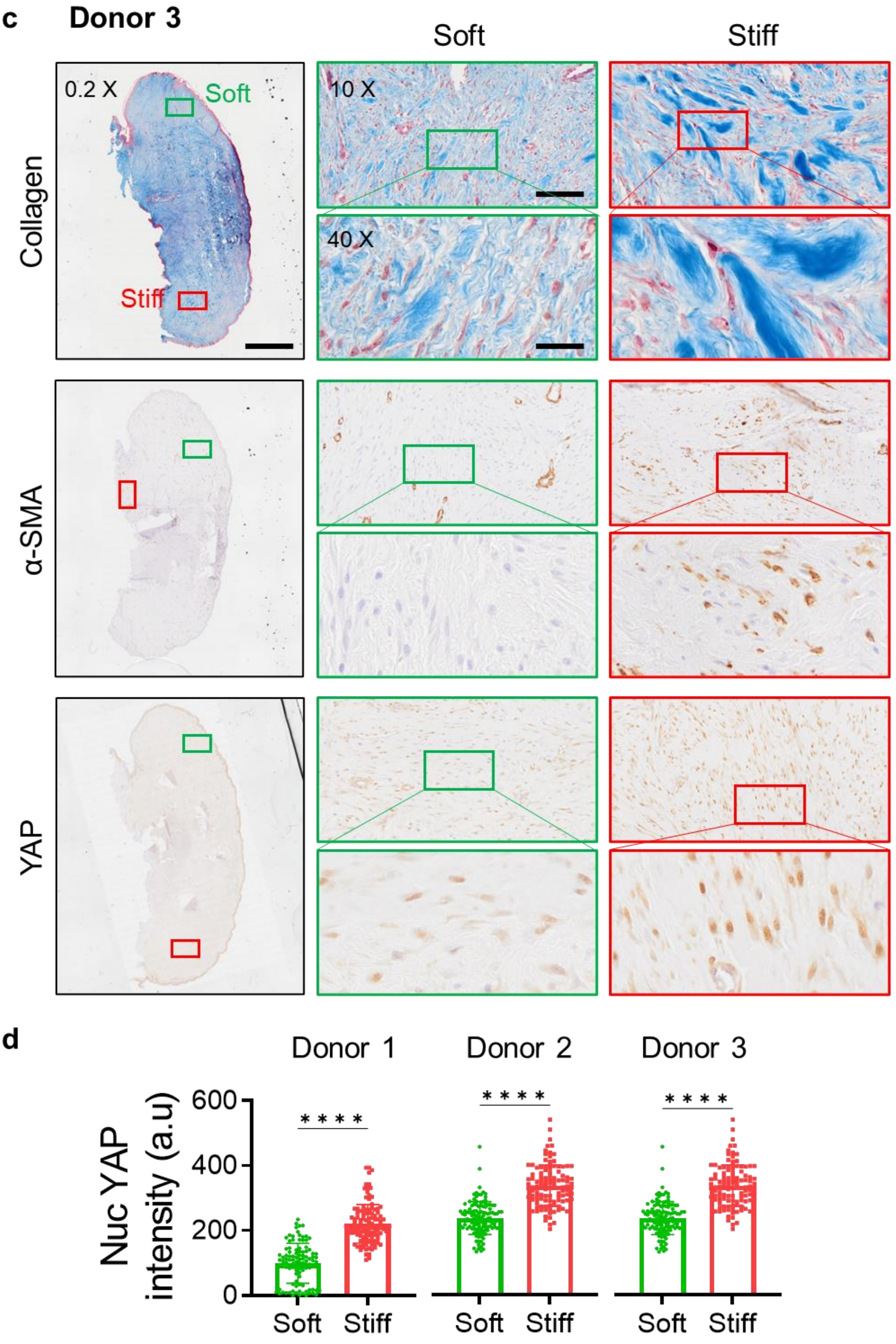

Supporting Information (Figs. S1-S24 &Table S1-S5)

Fig. S1. Histological analysis of human keloid tissues from three independent donors.

(a) Donor 1, **(b)** donor 2, and **(c)** donor 3. Masson's trichrome staining (top), IHC staining for α-SMA (middle), and YAP (bottom) in keloid tissues. Scale bar, 2 mm (0.2 X), 40 μm (10 X), and 10 μm (40 X). **(d)** Quantification of nuclear YAP intensity (n = 101-118 cells/donor). .****p < 0.0001; two-tailed paired Student's t-test.

Supporting Information (Figs. S1-S24 &Table S1-S5)

a

Blending (wt %)		Elastic modulus (kPa)
Sylgard 184	Sylgard 527	
0	100	10 ± 2
9	91	216 ± 17
17	83	519 ± 66
50	50	1432 ± 272
83	17	1685 ± 330
100	0	1910 ± 205

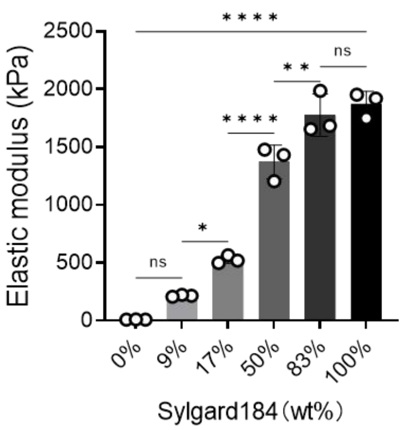

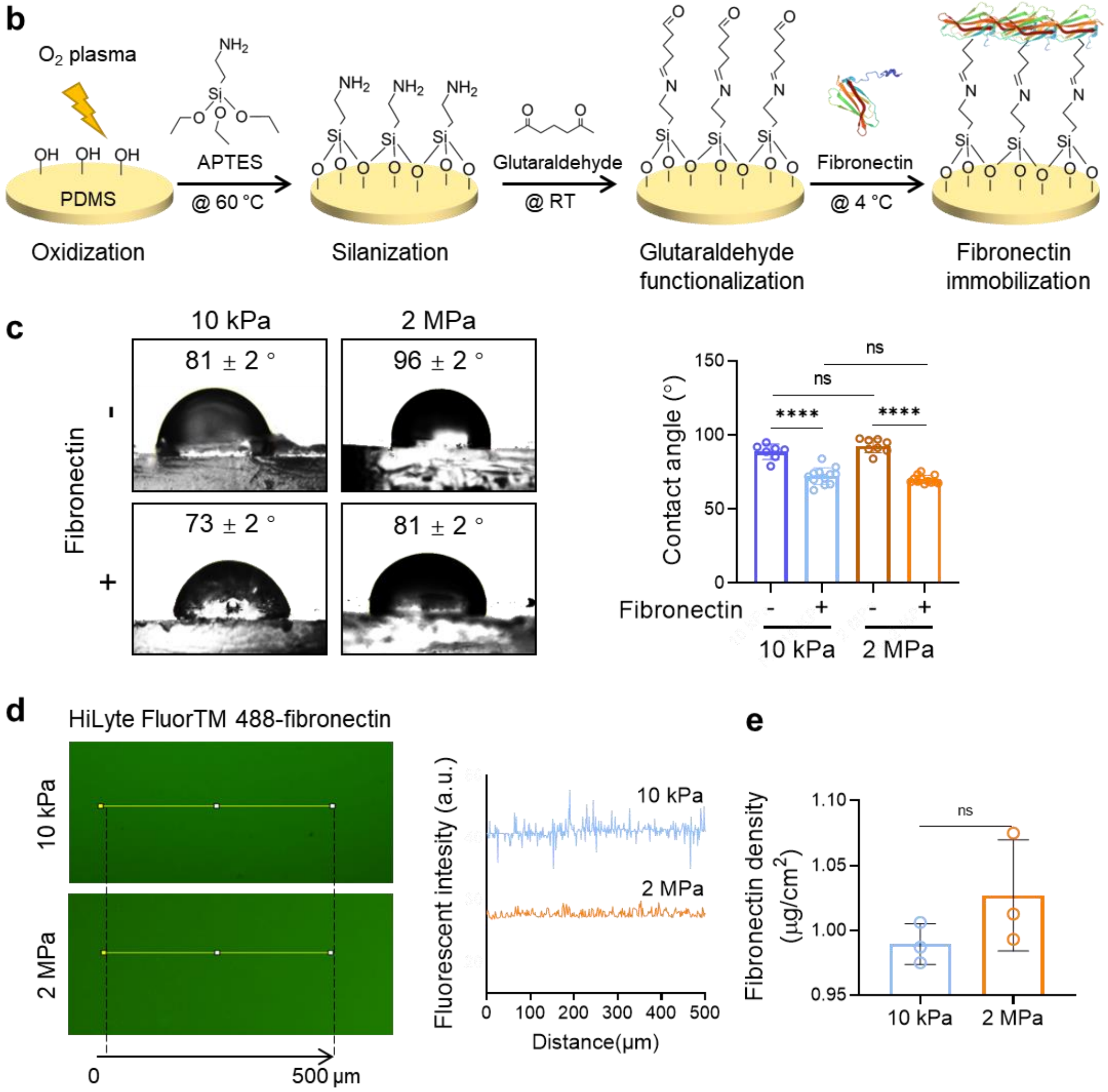

Fig. S2. Preparation of PDMS substrates.

(a) The blending ratios of Sylgard 184 and 527 and corresponding stiffness of PDMS substrates (n = 3). **(b)** Schematic illustrating the surface modification of PDMS substrates with

fibronectin. **(c)** Water contact angle measurement of fibronectin-immobilized PDMS substrates ($n = 7$-12). **(d, e)** Evaluation of the fibronectin immobilization on the substrates. Representative fluorescence images for substrates modified with HiLyte Fluor™ 488 labeled fibronectin (left) showing the homogenous distribution of fibronectin on the surface (right) (d). Yellow lines indicate the pixel regions used for intensity profiling. Quantification of the fibronectin density based on the fluorescence intensity showing the fibronectin immobilization amount is the same regardless of the substrate stiffness (e, $n = 3$). *$p < 0.05$, **$p < 0.01$, ***$p < 0.001$, and ****$p < 0.0001$; two-tailed paired Student's t-test or one-way ANOVA followed by Tukey's post hoc tests.

Supporting Information (Figs. S1-S24 &Table S1-S5)

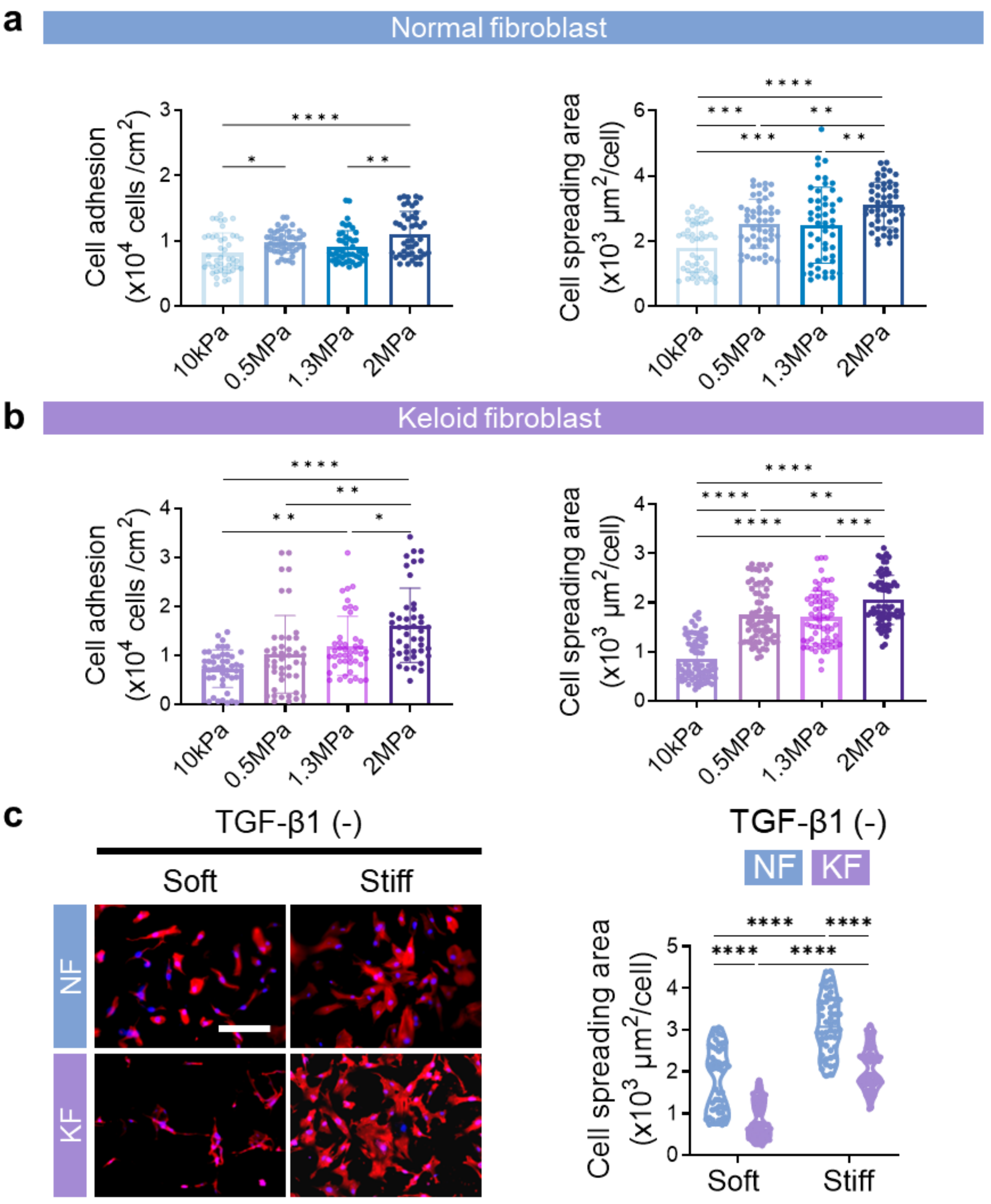

Fig. S3. Cell adhesion and spreading in response to the matrix stiffness after 2 days of culture on the substrates.

(a, b) Quantitative analysis of cell adhesion number (n = 45 cells/cm^2) and spreading area (n = 50-70 cells/condition) of normal fibroblast (NF, a) and keloid fibroblast (KF, b) in response to the stiffness of PDMS substrates. **(c)** Representative images of F-actin staining (red) of NF and KF cultured either soft (10 kPa) or stiff (2MPa) (left) along with the quantification of cell spreading area (right, n = 50-70 cells/condition). Scale bar, 200 μm. Nuclei are stained with DAPI (blue). Data are representative of at least three independent experiments. *p < 0.05, **p < 0.01, ***p < 0.001 and ****p < 0.0001; two-way ANOVA followed by Tukey's post hoc tests.

Supporting Information (Figs. S1-S24 &Table S1-S5)

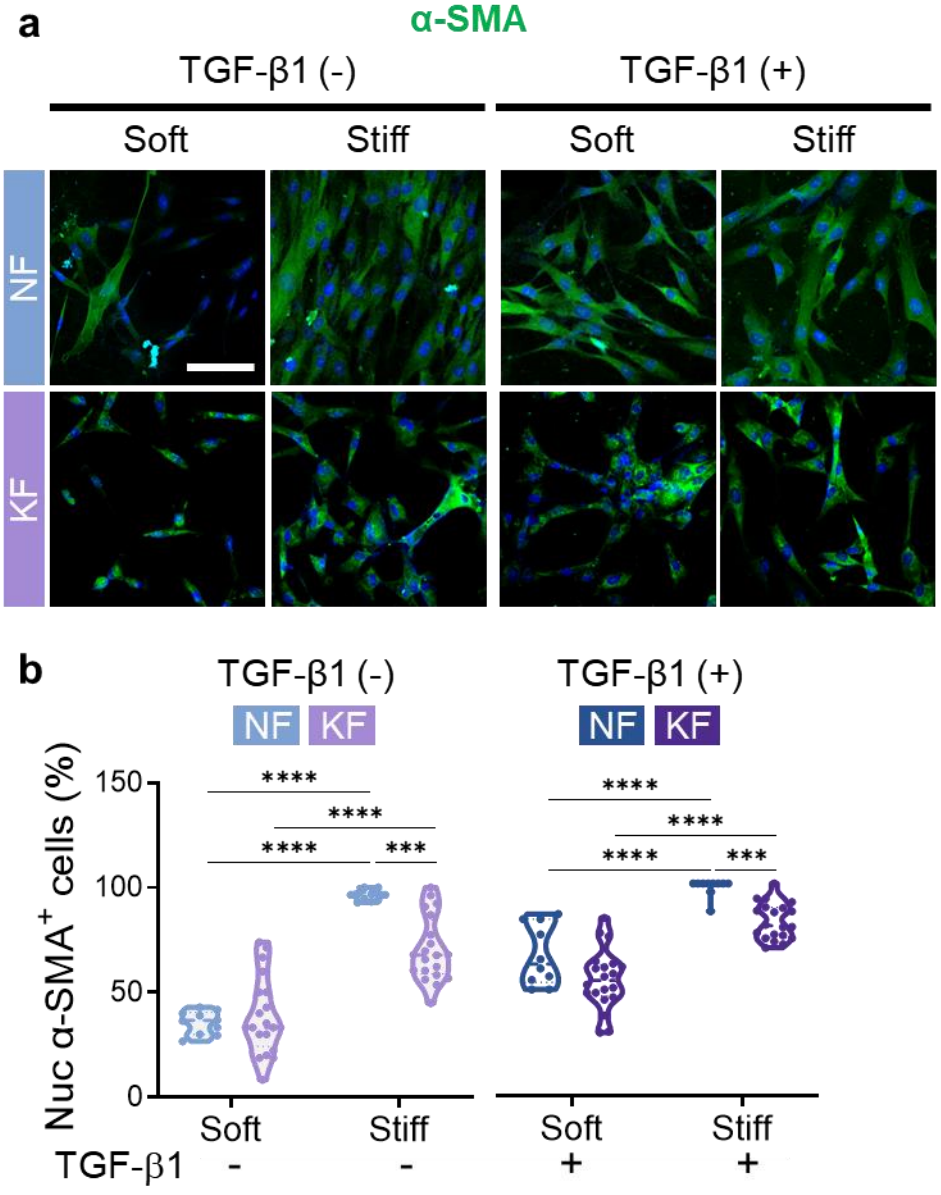

Fig. S4. α-SMA expression in normal fibroblasts and keloid fibroblasts after 2 days of culture on the substrates.

(a) Representative immunostaining for α-SMA (green) and **(b)** quantification of α-SMA-positive cells (%) (n = 10-20 fields/condition from 3 independent experiments). Scale bar, 100 μm. Nuclei are stained with DAPI (blue). **p < 0.01, ***p < 0.001 and ****p < 0.0001; two-way ANOVA followed by Tukey's post hoc tests.

Supporting Information (Figs. S1-S24 &Table S1-S5)

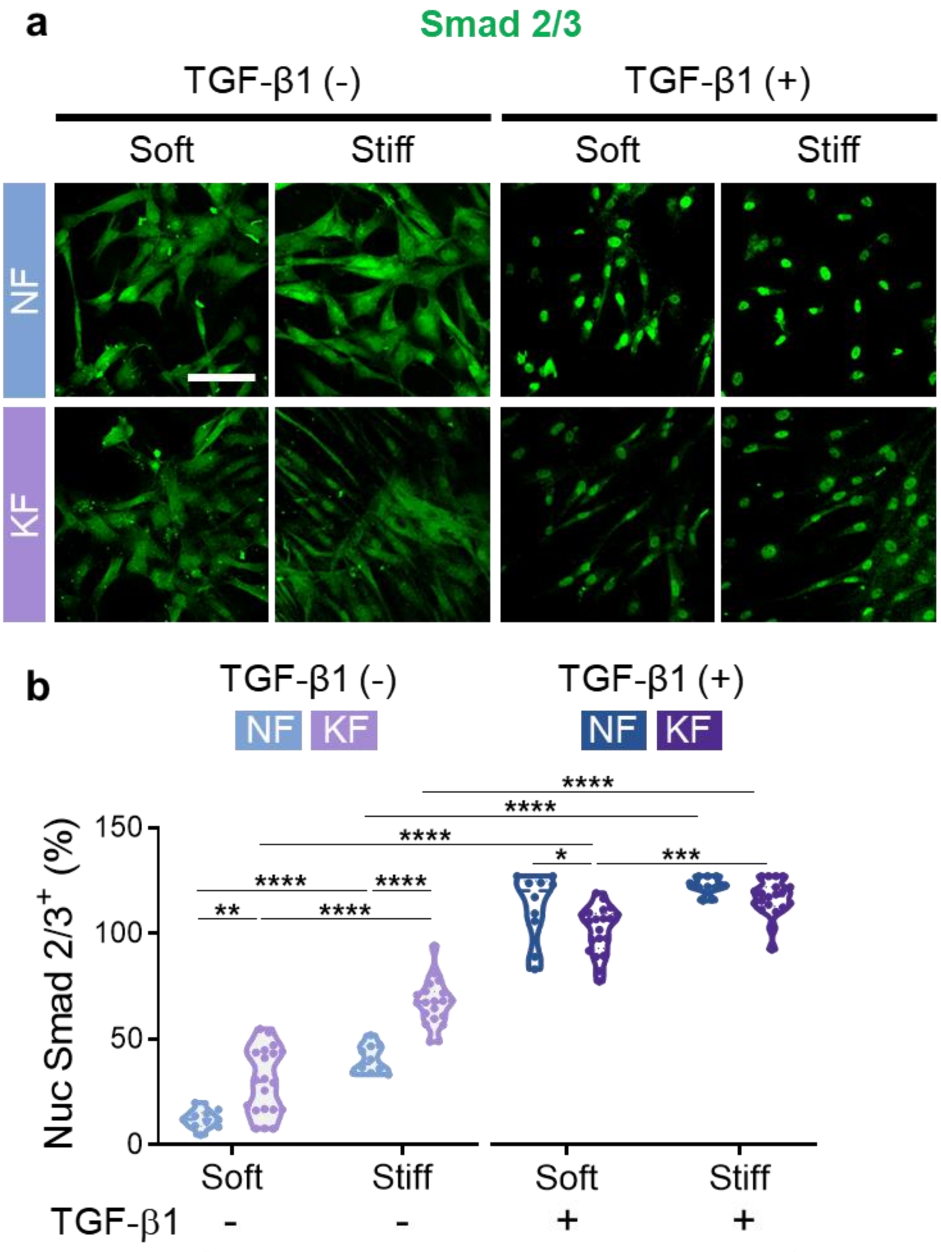

Fig. S5. Nuclear Smad2/3 localization in normal fibroblasts and keloid fibroblasts after 2 days of culture on the substrates.

(a) Representative immunostaining for Smad2/3 (green). Scale bar, 100 μm. (**b)** Quantitative analysis of nuclear MRTF-A positive cells (n = 10 - 20 fields/condition from 2 independent experiments). *p < 0.05, **p < 0.01, ***p < 0.001, and ****p < 0.0001; two-way ANOVA followed by Tukey's post hoc tests.

Supporting Information (Figs. S1-S24 &Table S1-S5)

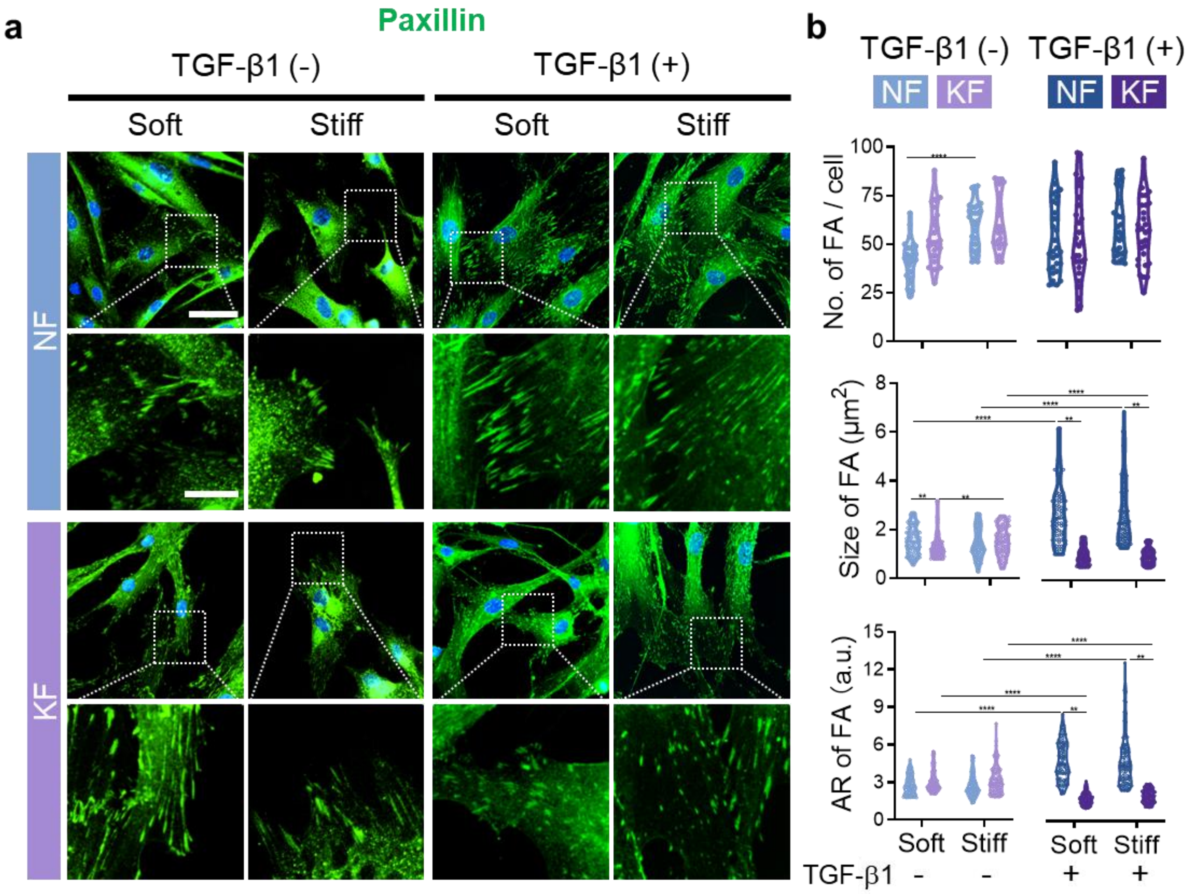

Fig. S6. Focal adhesion formation in normal fibroblasts and keloid fibroblasts after 2 days of culture on the substrates.

(a) Representative immunostaining for paxillin (green) and **(b)** quantitative analysis of focal adhesion (FA) formations including the quantification of the number (n = 30 FA number/cell), size (n = 100 FA/condition), and aspect ratio (AR, n = 100 FA/condition) of focal adhesions based on the immunostaining images. Data are representative of at least three independent experiments. Scale bar, 50 μm (main images), and 15 (inserts). Nuclei are stained with DAPI (blue). $^{**}p < 0.01$ and $^{****}p < 0.0001$; two-way ANOVA followed by Tukey's post hoc tests.

Supporting Information (Figs. S1-S24 &Table S1-S5)

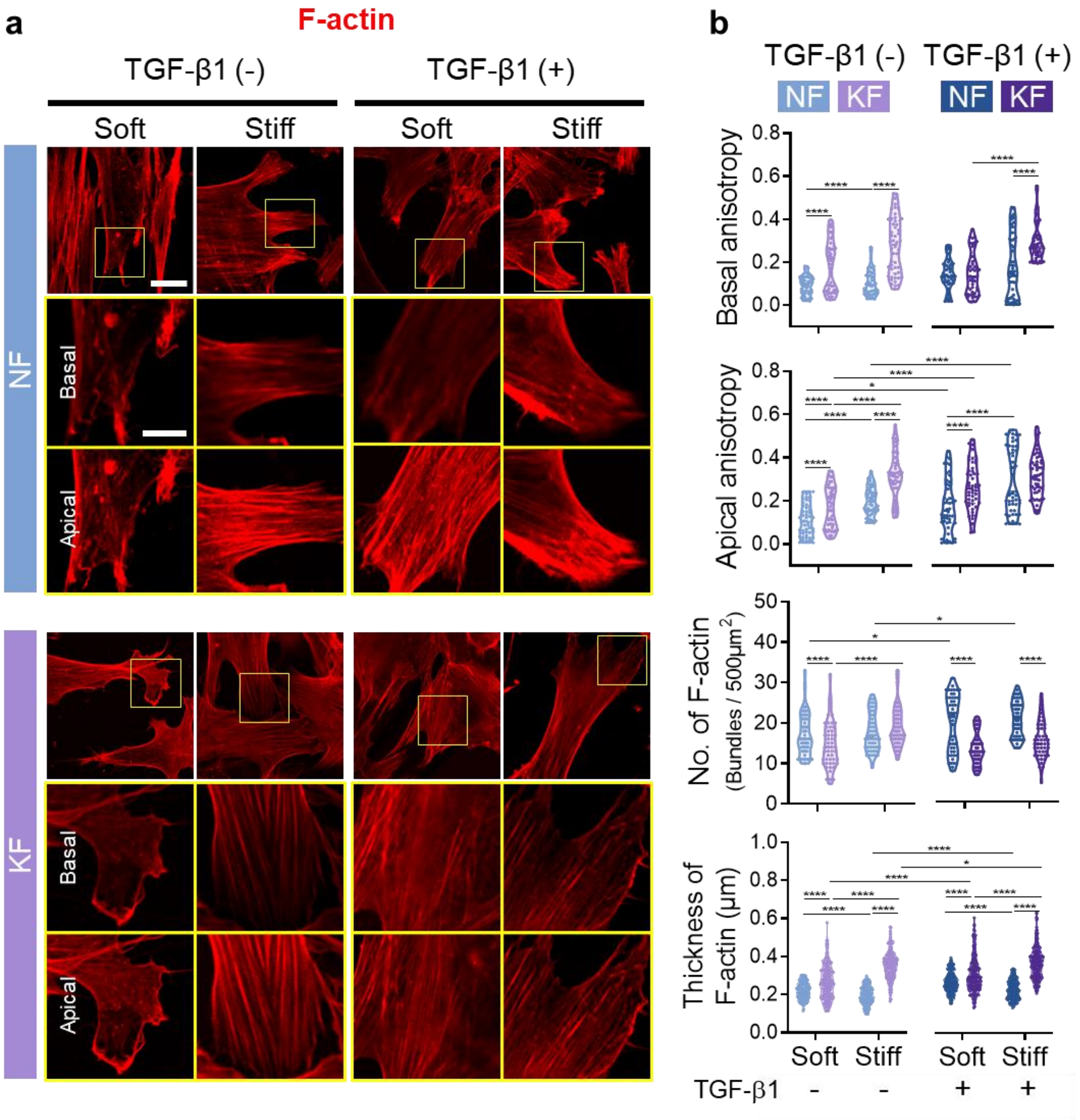

Fig. S7. Actin formation in normal fibroblasts and keloid fibroblasts after 2 days of culture on the substrates.

(a) Representative images of F-actin staining (red) on the basal and apical planes and **(b)** quantitative analysis of the F-actin anisotropy, the number, and thickness of F-actin bundles (d, n = 50 - 200 cells/condition from 3 independent experiments). Scale bar, 25 μm (main images), and 10 μm (inserts). *p < 0.05 and ****p < 0.0001; two-way ANOVA followed by Tukey's post hoc tests.

Supporting Information (Figs. S1-S24 &Table S1-S5)

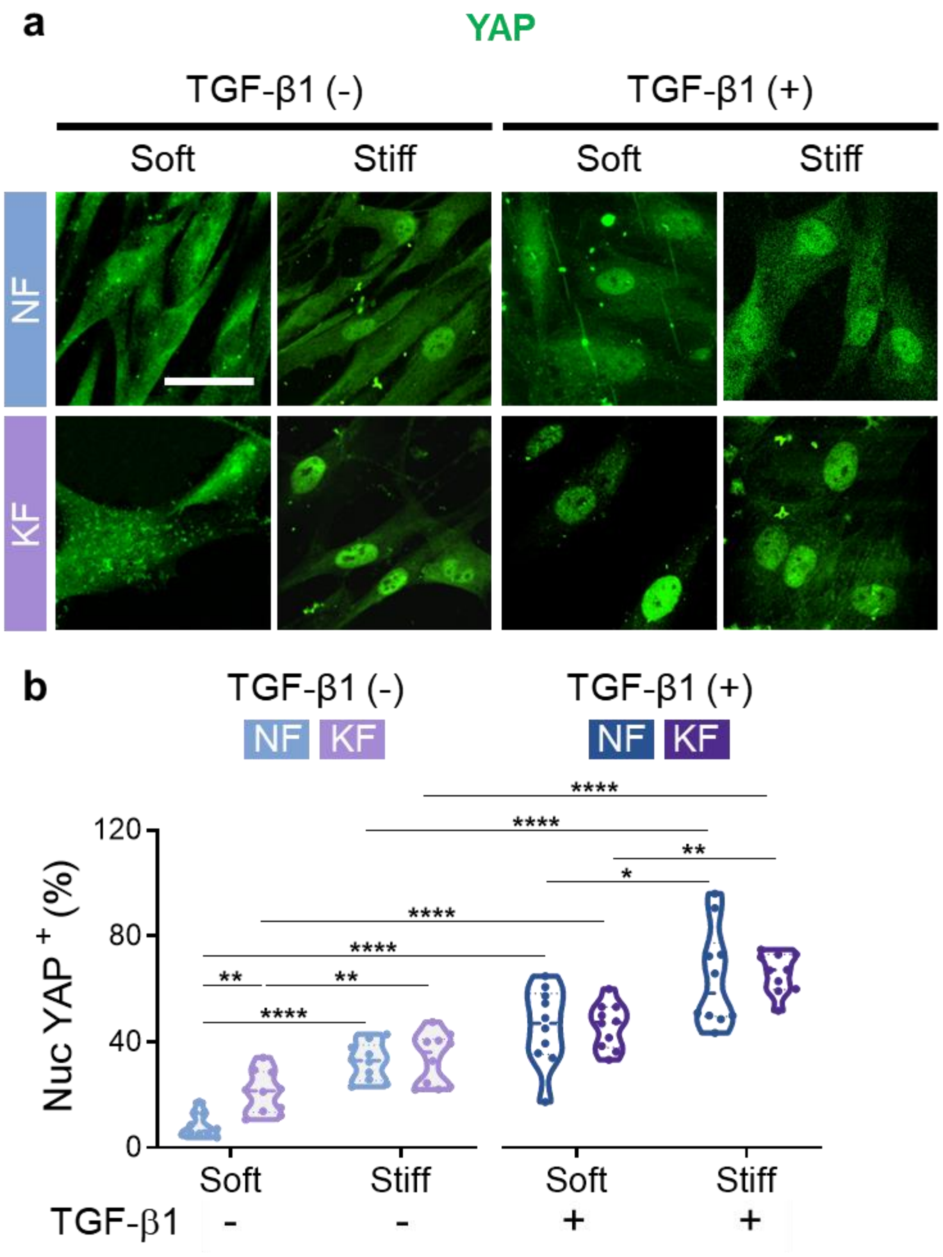

Fig. S8. Nuclear YAP localization in normal fibroblasts and keloid fibroblasts after 2 days of culture on the substrates.

(a) Representative immunostaining for YAP (green). Scale bar, 40 μm. (**b)** Quantitative analysis of nuclear YAP positive cells (n = 10 fields/condition from 3 independent experiments). *$p < 0.05$, **$p < 0.01$ and ****$p < 0.0001$; two-way ANOVA followed by Tukey's post hoc tests.

Supporting Information (Figs. S1-S24 &Table S1-S5)

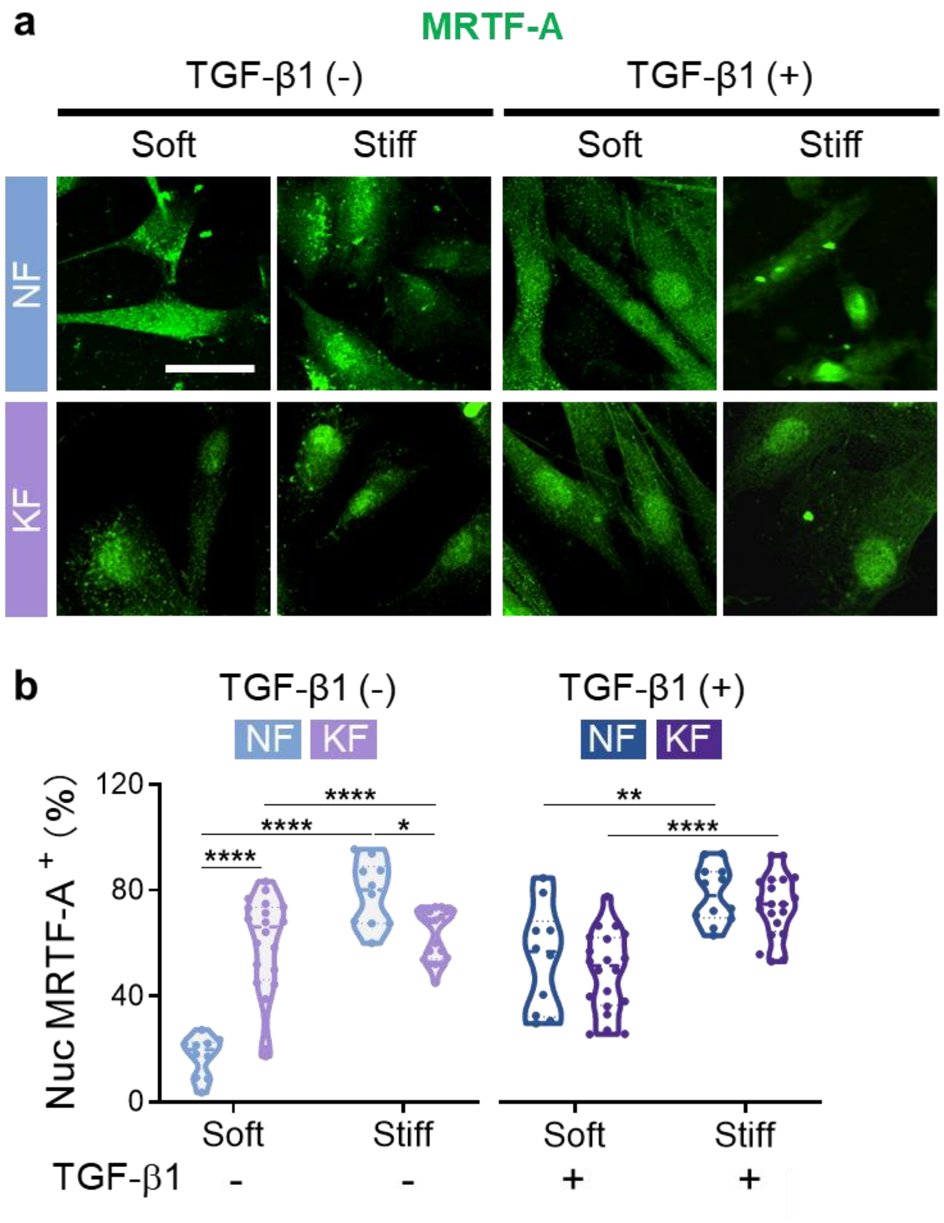

Fig. S9. Nuclear MRTF-A localization in normal fibroblasts and keloid fibroblasts after 2 days of culture on the substrates.

(a) Representative immunostaining for MRTF-A (green). Scale bar, 40 μm. (**b)** Quantitative analysis of nuclear MRTF-A positive cells (n = 10-20 fields/condition from 2 independent experiments). *p < 0.05, **p < 0.01 and ****p < 0.0001; two-way ANOVA followed by Tukey's post hoc tests.

Supporting Information (Figs. S1-S24 &Table S1-S5)

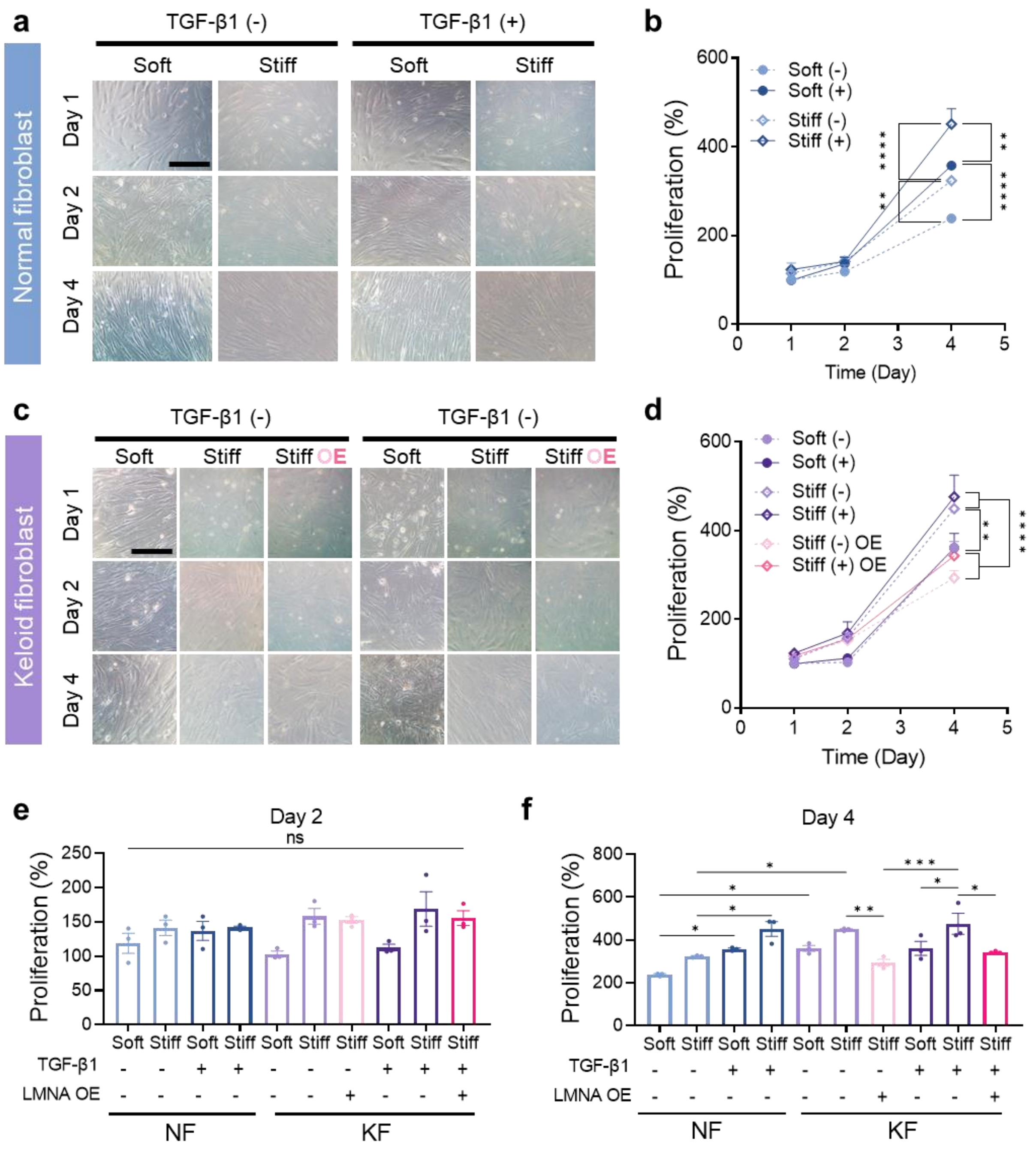

Fig. S10. Evaluation of in vitro cell proliferation.

NFs or KFs were cultured on either soft or stiff substrates with or without TGF-β1 treatment for 4 days. **(a-d)** Representative bright-field images of NFs (a) or KFs (c) cultured on soft or stiff substrates with or without TGF-β1 treatment after 1, 2, and 4 days of culture (left) and quantification of the proliferation rate based on CCK-8 assay (b, d, n = 3 replicates/condition from 3 independent experiments). Scale bar, 500 μm. **(e, f)** Bar graphs representing the proliferation rate at day 2 (e) or day 4 after (f) culture. *p < 0.05, **p < 0.01, ***p < 0.001, ****p < 0.0001 and *ns*: not significant; two-way ANOVA followed by Tukey's post hoc tests.

Supporting Information (Figs. S1-S24 &Table S1-S5)

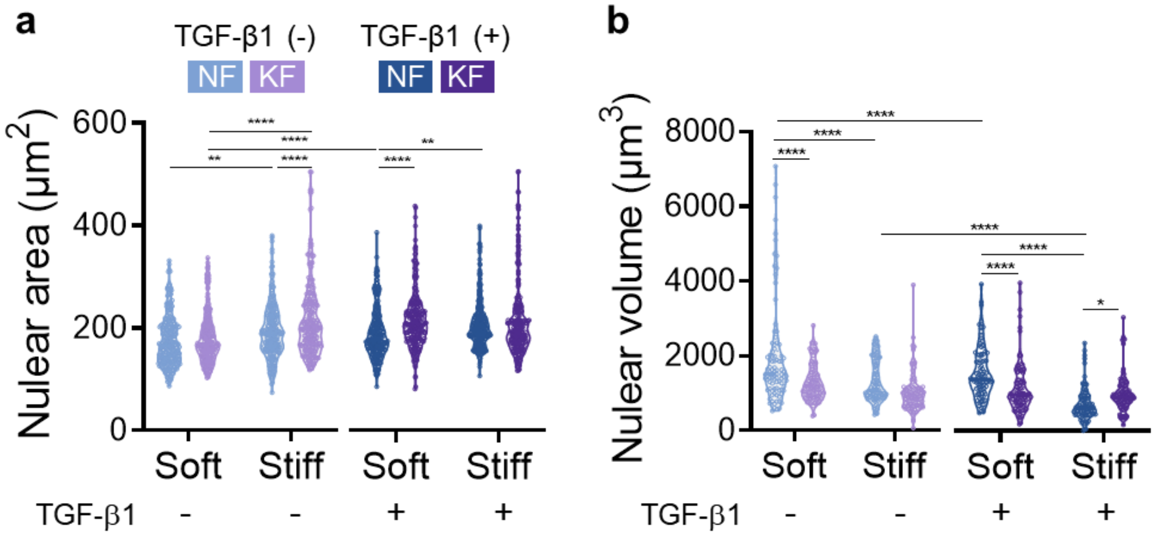

Fig. S11. Nuclear morphology analysis.

(a) Quantitative analysis of nuclear area (n = 260 nuclei/condition) and volume (b, n = 90 - 100 nuclei/condition). Data are representative of at least three independent experiments. *$p < 0.05$, **$p < 0.01$ and ****$p < 0.0001$; two-way ANOVA followed by Tukey's post hoc tests.

Supporting Information (Figs. S1-S24 &Table S1-S5)

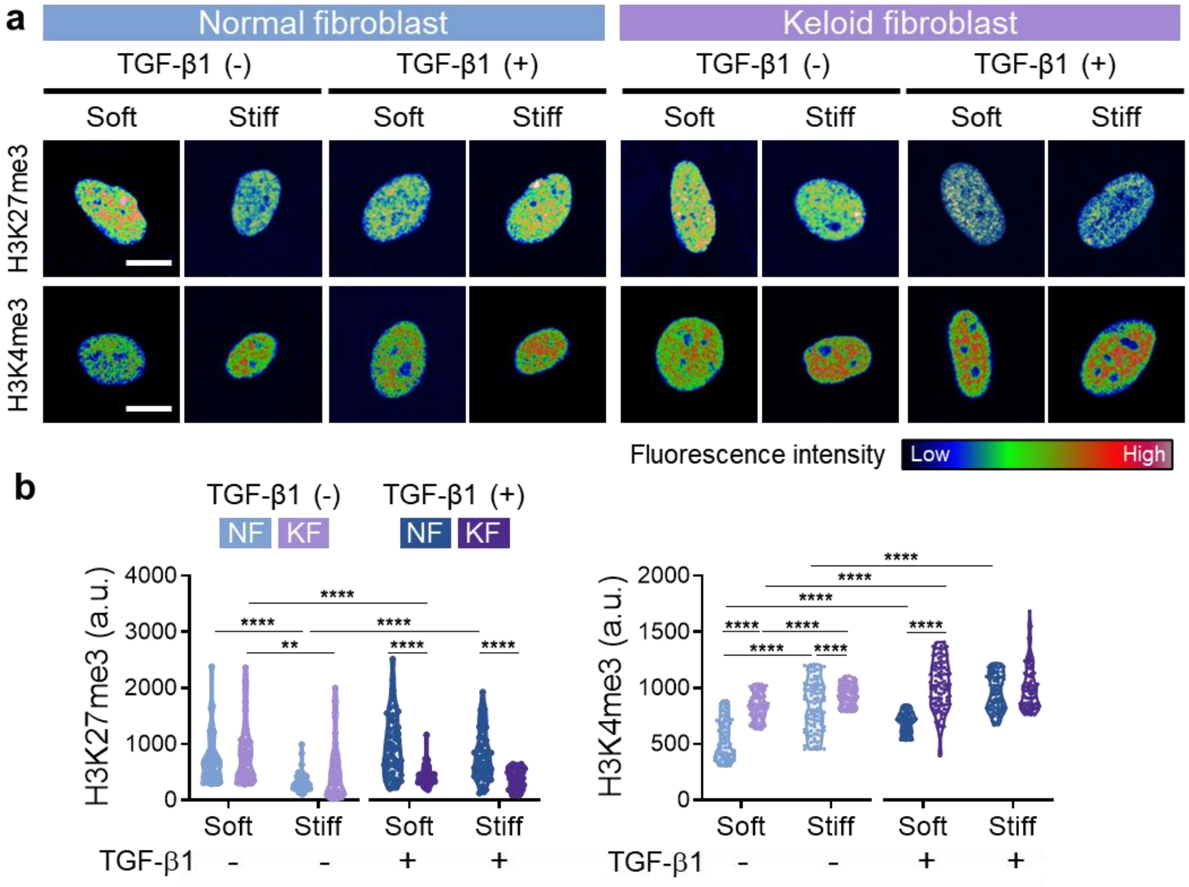

Fig. S12. Immunostaining for histone methylation.

(a) Representative color-coded images of H3K27 methylation (top) and H3K4 methylation (bottom). Scale bar, 10 μm. (**b)** Mean intensity of the immunostaining for H3K27me3 (left, n = 50 nuclei/condition) and H3K4me3 (right, n = 100 nuclei/condition). Data are representative of at least three independent experiments. **$p < 0.01$ and ****$p < 0.0001$; two-way ANOVA followed by Tukey's post hoc tests.

Supporting Information (Figs. S1-S24 &Table S1-S5)

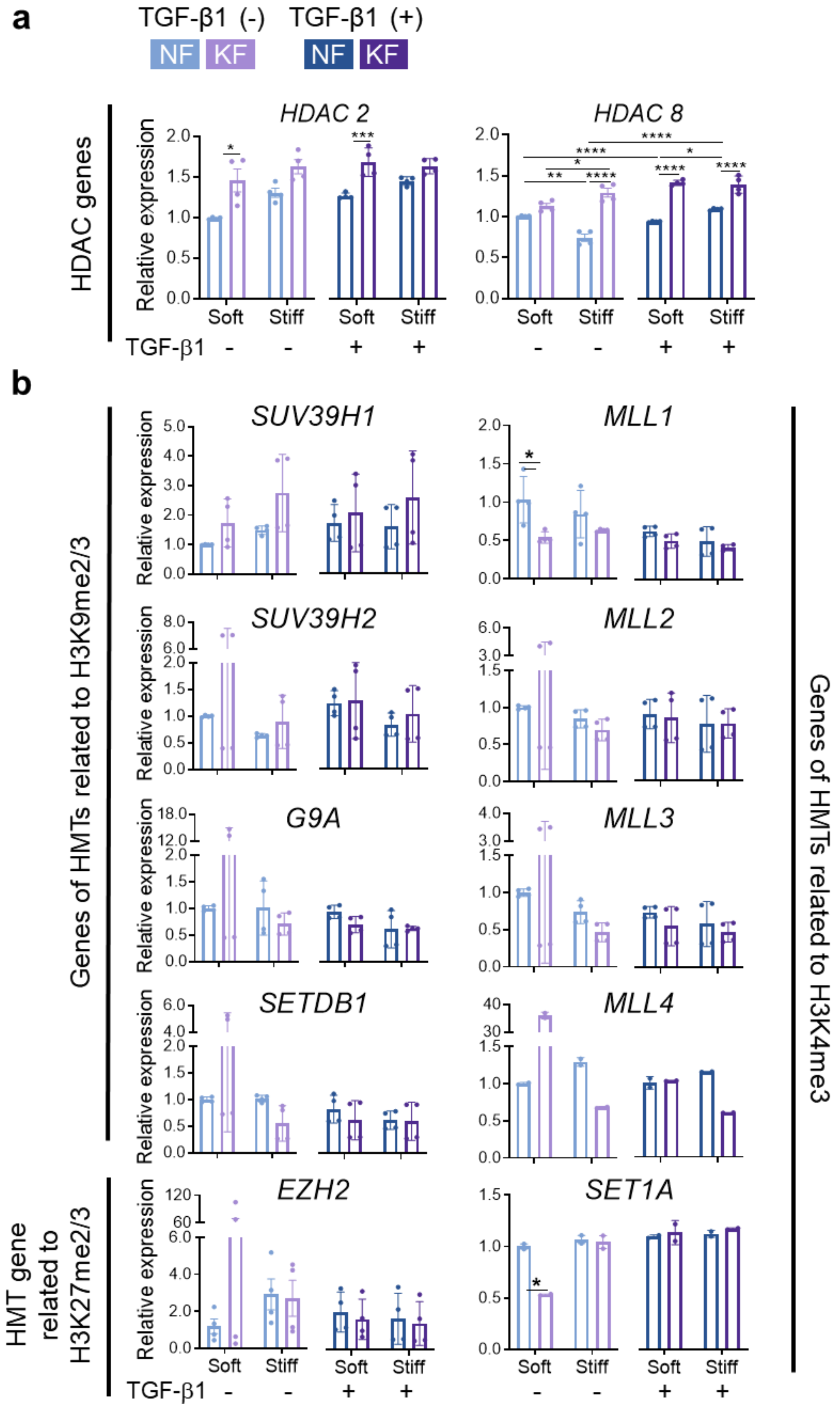

Fig. S13. Gene expression levels of histone methyltransferases (HMTs) and histone deacetylases (HDACs) in fibroblasts after 2 days of culture on the substrates.

(a) Genes of HDACs related to histone acetylation (i.e., HDAC2 and HDAC8) and **(b)** genes of HMTs related to H3K9me3 (i.e., Suv39H 1, Suv39H2, G9a, and STEB1), H3K27me3 (i.e., Ezh2), and H3K4me3 (i.e., MLL1, MLL2, MLL3, MLL4, and SET1A) quantified by qRT-PCR (n = 2 - 4 replicates from 2 - 3 independent experiments). *p < 0.05, **p < 0.01, ***p < 0.001, and ****p < 0.0001; two-way ANOVA followed by Tukey's post hoc tests.

Supporting Information (Figs. S1-S24 &Table S1-S5)

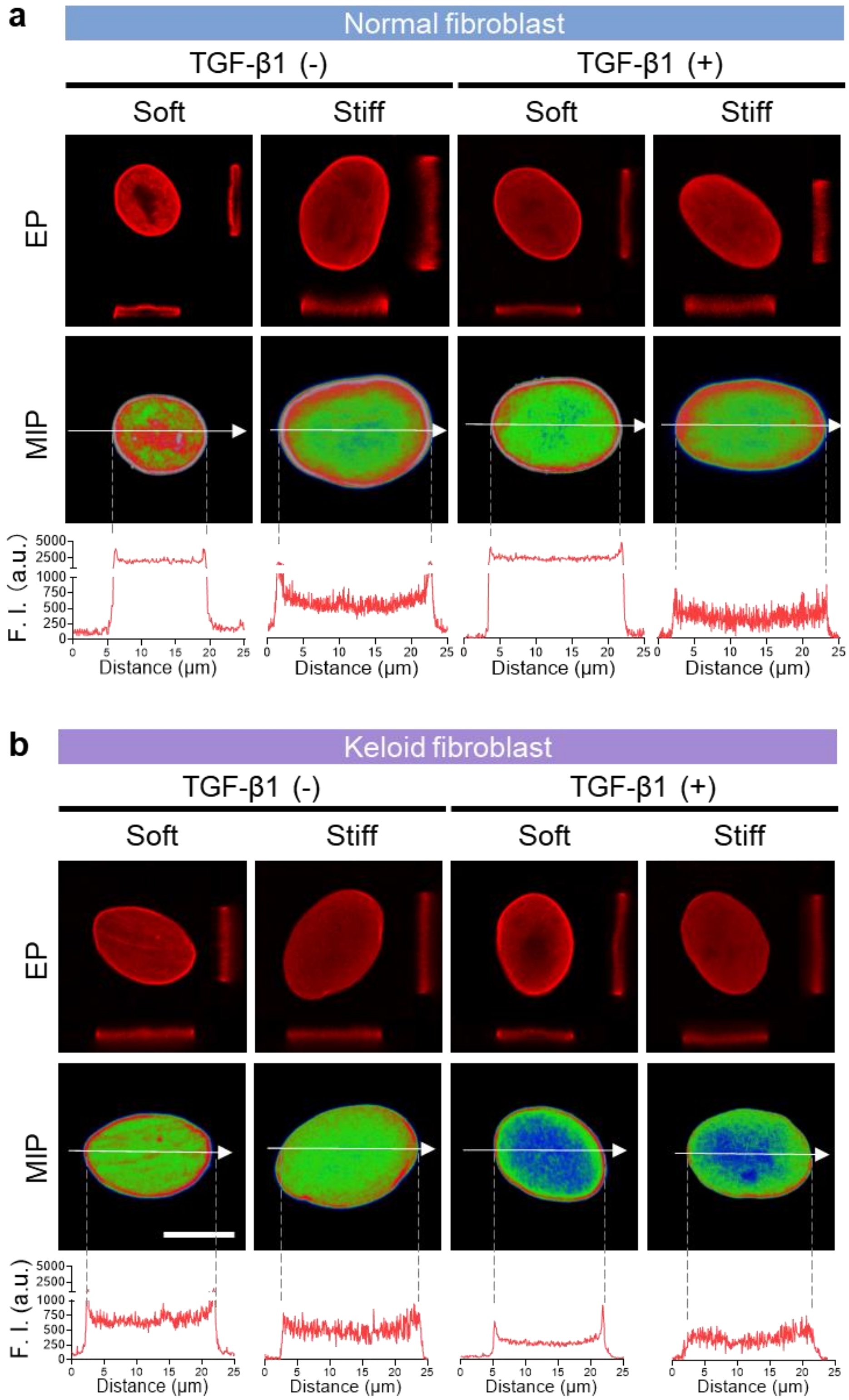

Fig. S14. Lamin A/C immunostaining showing its distribution within the nucleus of normal fibroblasts (a) or keloid fibroblasts (b).

XZ- and YZ-axes projections on the equatorial plane (EP) of the nucleus (top). Maximum intensity projection (MIP) of a series of z-stacks images covering the entire nuclear region

(middle), along with line scans of fluorescence intensity (F. I.) from the immunostaining images (bottom). Scale bar, 10 μm. Dotted lines mark the edges of the nucleus.

Supporting Information (Figs. S1-S24 &Table S1-S5)

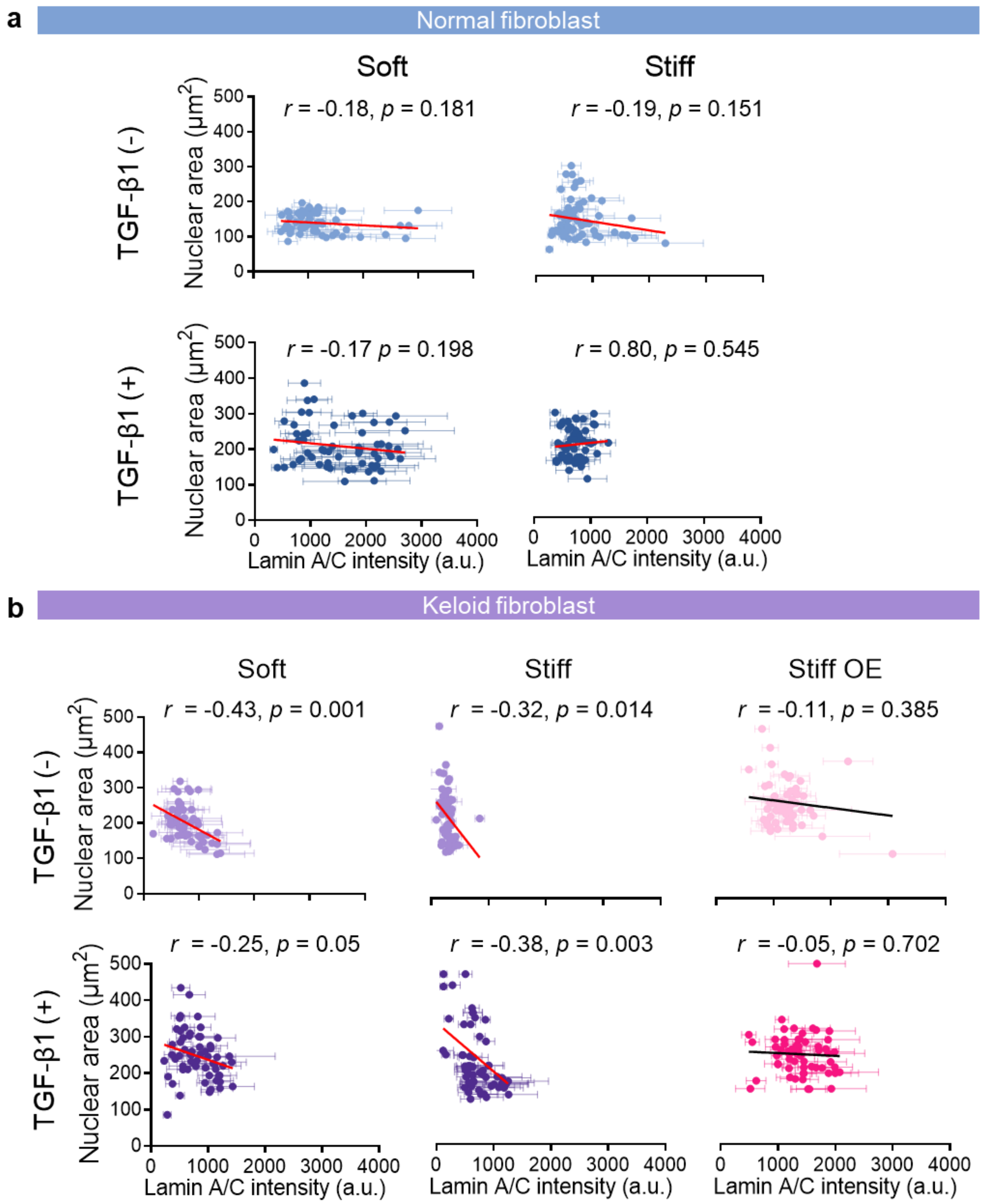

Fig. S15. Correlation plots depicting the relationship between nuclear size and lamin A/C intensity (n = 60 nuclei from 3 independent experiments).

The correlation coefficient (Pearson's *r*) was determined by linear fits as in solid lines.

Supporting Information (Figs. S1-S24 &Table S1-S5)

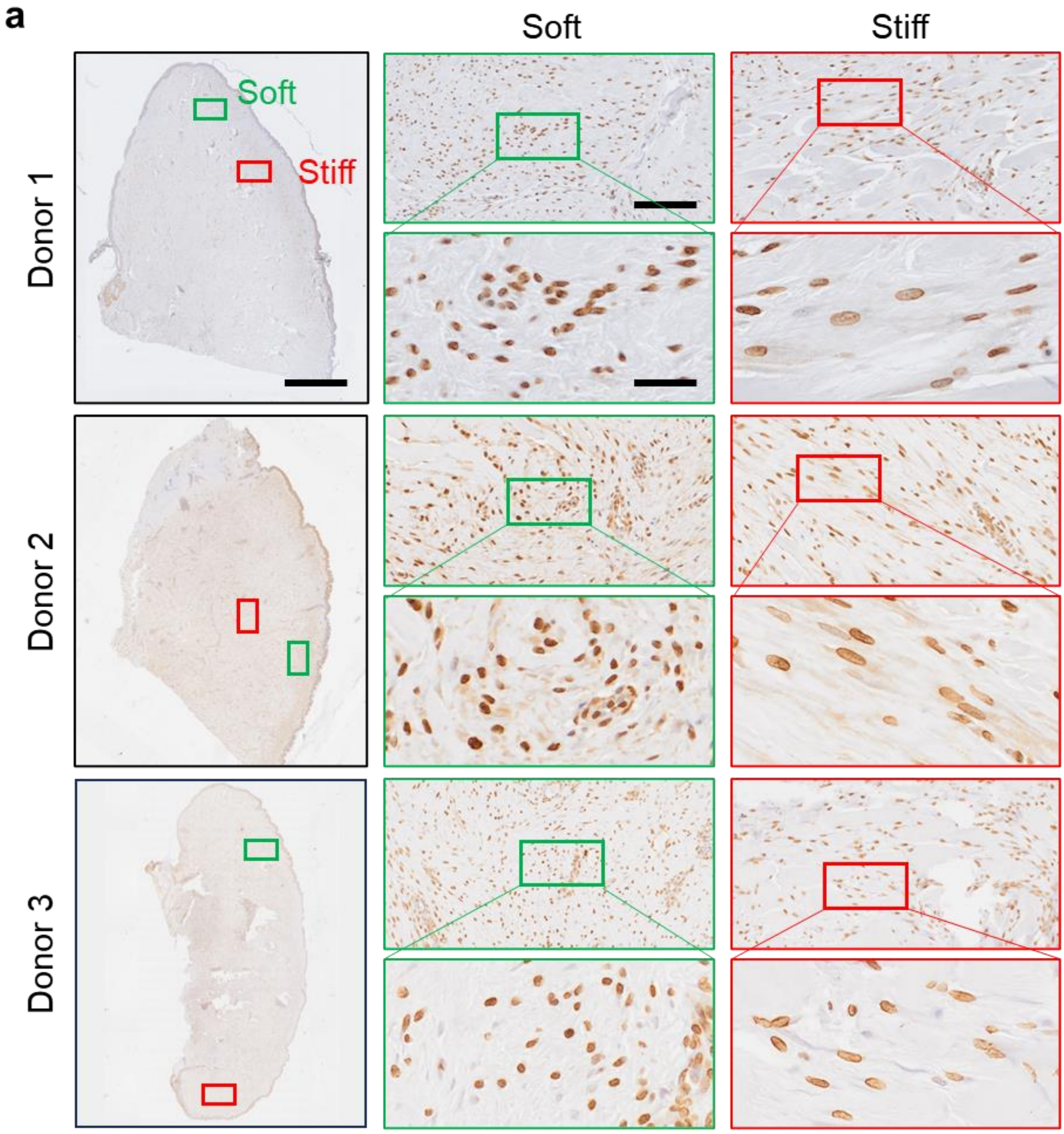

Supporting Information (Figs. S1-S24 &Table S1-S5)

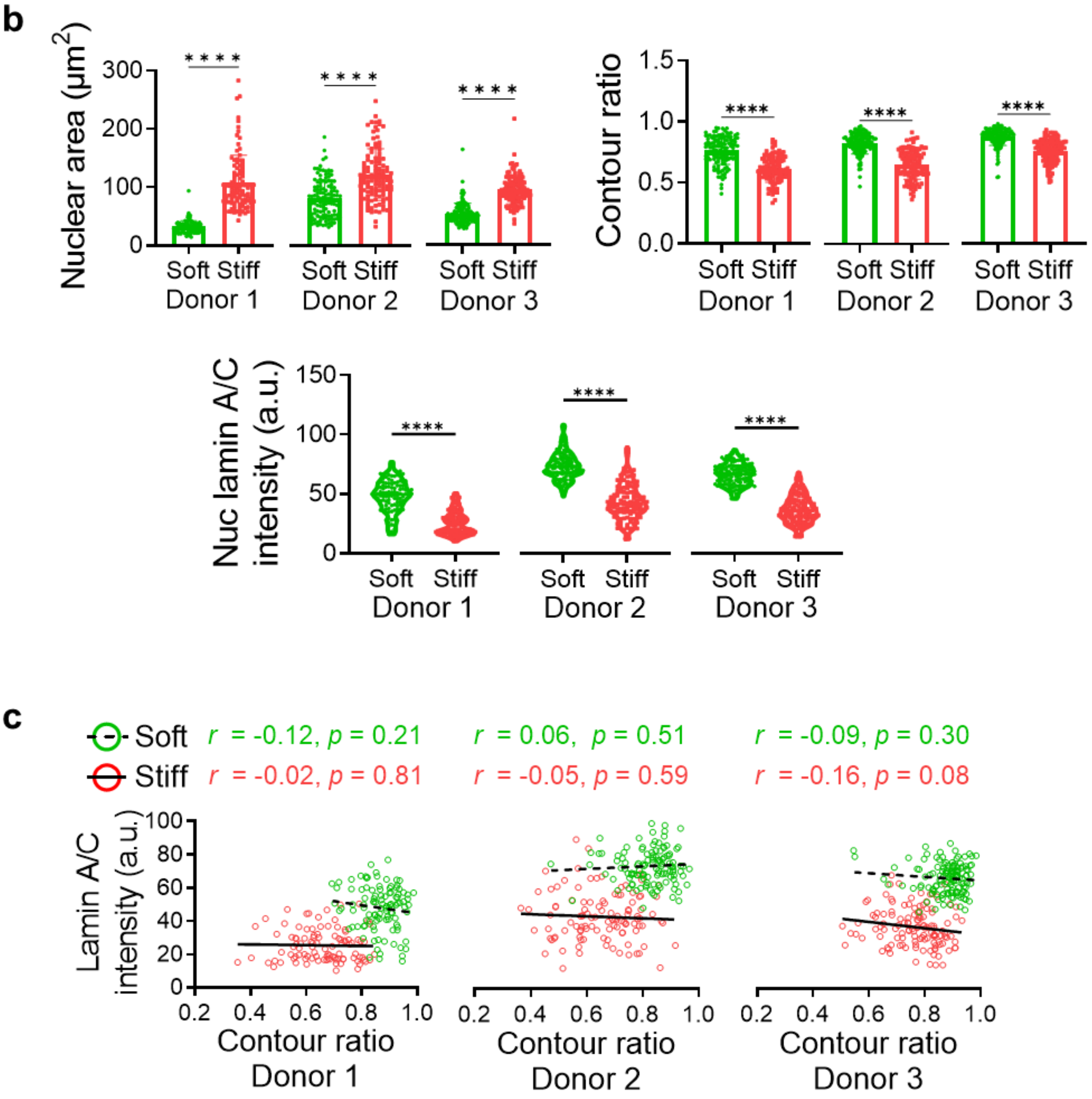

Supporting Information (Figs. S1-S24 &Table S1-S5)

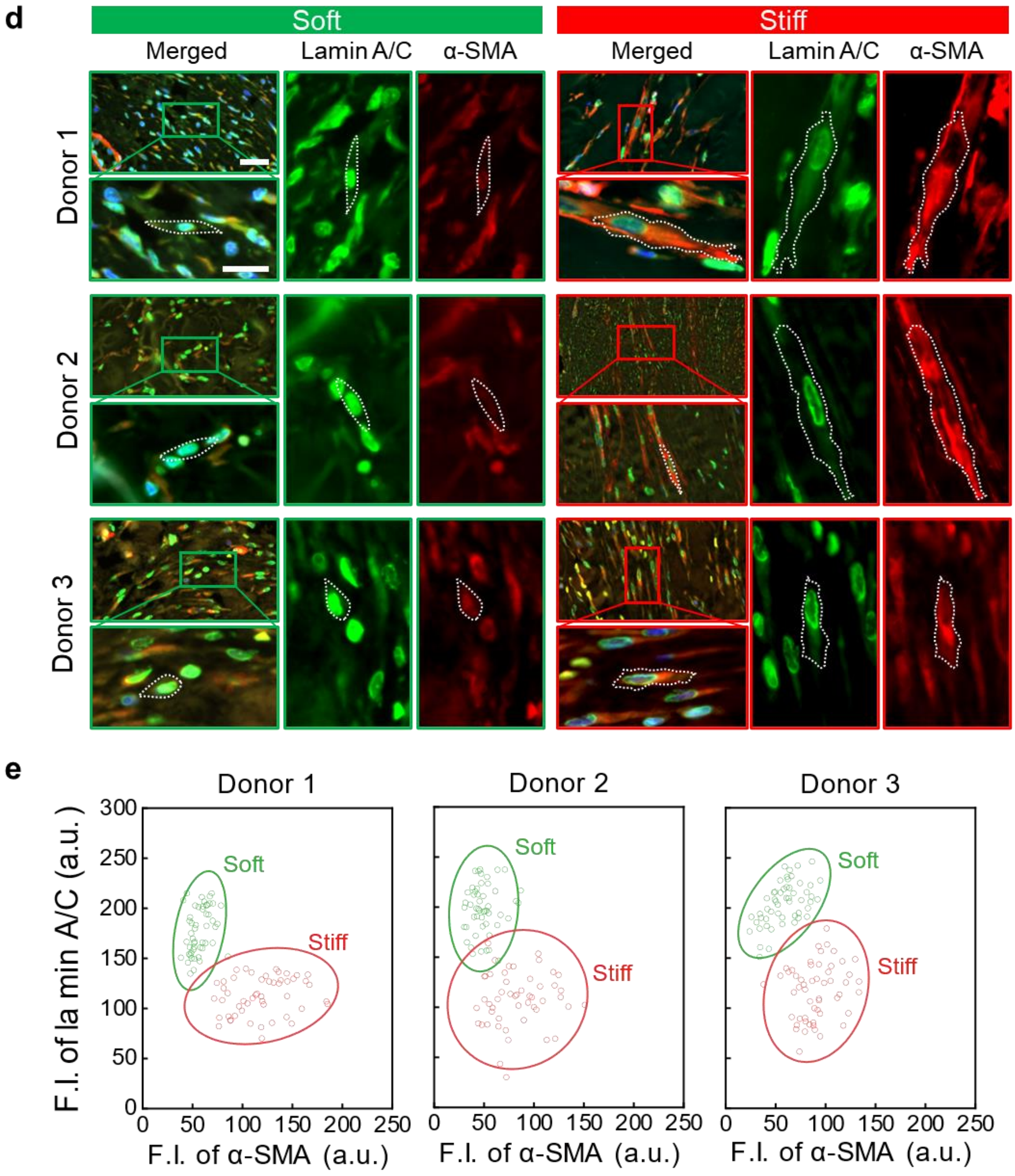

Fig. S16. Lamin A/C expression in human keloid tissues from three independent donors.

(a) Representative images of immunohistochemical staining for lamin A/C in human keloid tissues. Scale bar, 2 mm (0.2 X), 40 μm (10 X), and 10 μm (40 X). **(b)** The nuclear area (n = 100 – 144 nuclei/condition), contour ratio (n = 100 - 136 nuclei/condition), and lamin A/C intensity (n = 100 - 135 nuclei/condition) of nuclei quantified based on the immunostaining. **(c)** Correlation plots depicting the relationship between lamin A/C intensity and contour ratio. The correlation coefficient (Pearson's *r*) was determined by linear fits as in (c, dash line for Soft, and solid line for Stiff). (d) Representative images of co-immunostaining for lamin A/C (green)

and a-SMA (red) in human keloid tissues. Scale bar, 10 µm (main) and 50 µm (insert). (e) Cluster Map displaying the correlation of lamin A/C and α-SMA intensity in soft (green) and stiff (red) regions (n = 50 – 54 cells/condition). Data represent mean ± s.d. of *n* and are representative of at least three independent experiments. *p < 0.05, **p < 0.01, ***p < 0.001 and ****p < 0.0001; one-way ANOVA followed by Tukey's post hoc tests.

Supporting Information (Figs. S1-S24 &Table S1-S5)

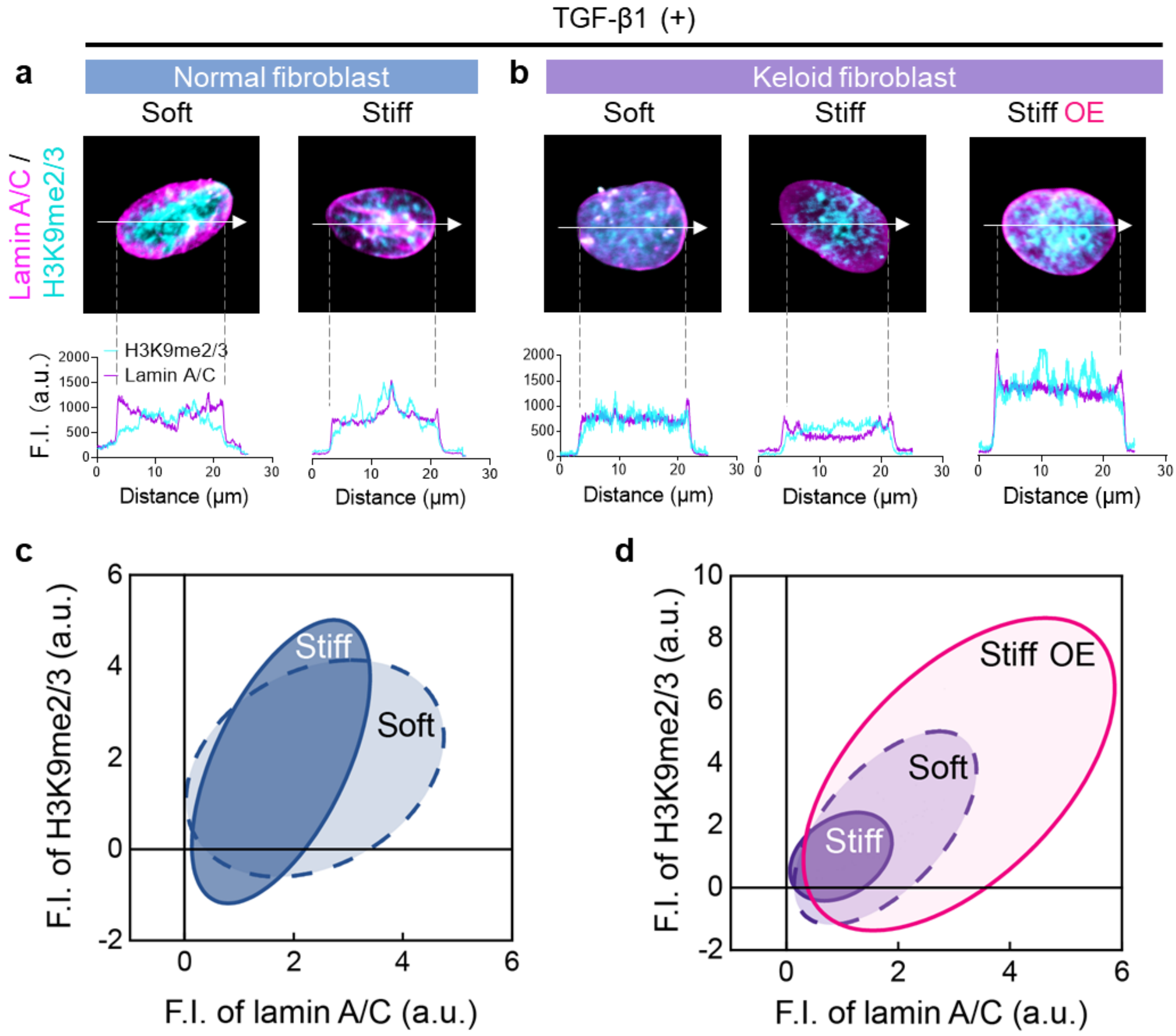

Fig. S17. Lamina-associated chromatin perinuclear anchoring.

(a) Representative images of co-immunostaining for lamin A/C and H3K9me2/3 (top) along with line scans of fluorescence intensity (F. I.) from the immunostainings (bottom). **(b)** Cluster Map displaying the correlation of lamin A/C and H3K9me2/3 intensity in each condition (n = 50 nuclei from 2 independent experiments). OE, lamin A/C-overexpressed keloid fibroblasts.

Supporting Information (Figs. S1-S24 &Table S1-S5)

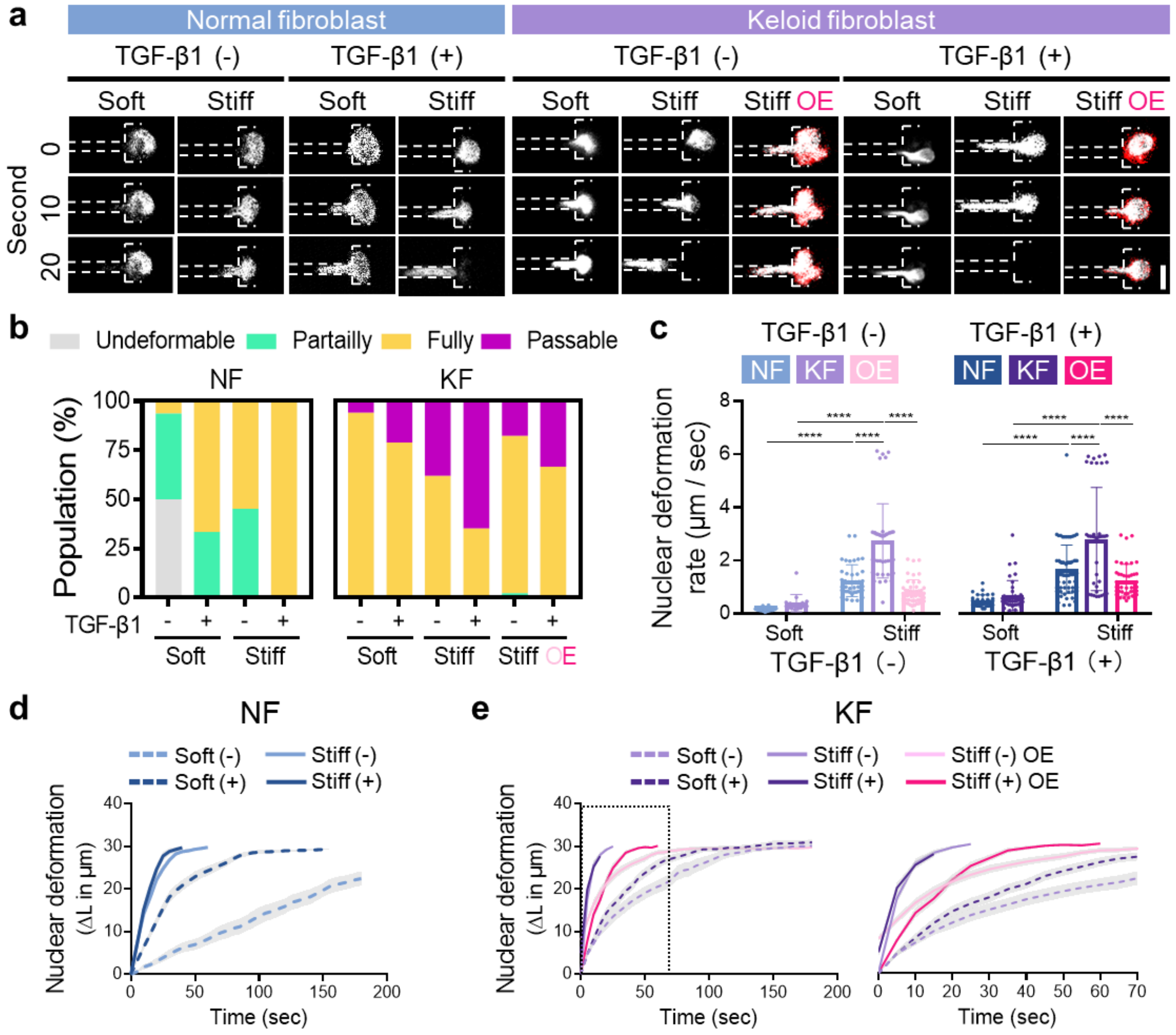

Condition		Slope	r^2
NF	Soft (-)	0.16	0.57
	Soft (+)	0.62	0.58
	Stiff (-)	1.08	0.66
	Stiff (+)	1.27	0.75

Condition		Slope	r^2
KF	Soft (-)	0.14	0.65
	Soft (+)	0.13	0.50
	Stiff (-)	1.07	0.65
	Stiff (+)	1.47	0.55
	Stiff (-) OE	0.07	0.34
	Stiff (+) OE	0.45	0.64

Fig. S18. Nuclear deformability analysis by the microfluidic micropipette aspiration assay.

(a) Representative time-lapse image series of the deformation of fluorescently labeled cell nuclei in the microfluidic micropipette aspiration device. Scale bar, 10 µm. NFs, KFs, or KFs transfected with mCherry-LMNA plasmid (OE) were pre-cultured on either soft or stiff substrates for 2 days before the assay. **(b)** The relative proportions of populations depending on nuclear deformability. **(c)** The nuclear deformation rate was measured based on the protrusion length upon aspiration (n > 30 nuclei/condition). **(d, e)** Nuclear protrusion profiles of NF (d) and KF (e) for 180 sec of aspiration (d, n = 22 – 45 nuclei/condition). The slope (*m*)

Supporting Information (Figs. S1-S24 &Table S1-S5)

and R squared (r^2) values were determined by linear fits as shown in (d, e).

Data represent mean ± s.d. (b) or mean ± s.e.m. (c) of *n* and are representative of at least three independent experiments. ****$p < 0.0001$; one-way ANOVA followed by Tukey's post hoc tests.

Supporting Information (Figs. S1-S24 &Table S1-S5)

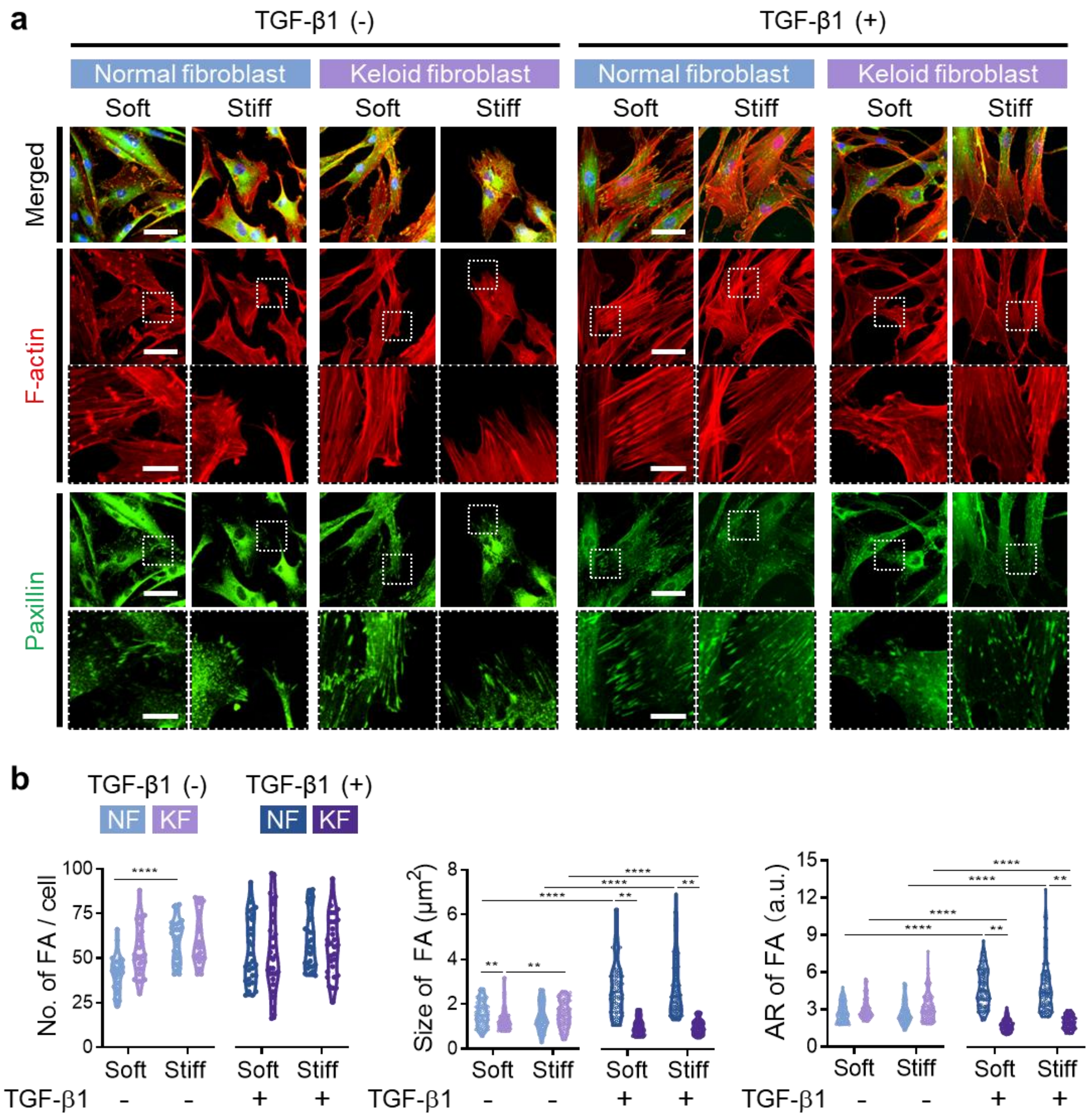

Fig. S19. TGF-β1 treatment reduces focal adhesion formation in keloid fibroblasts.

(a) Representative co-staining images of paxillin (green) and F-actin (red). Scale bar, 50 μm (main images), and 15 μm (inserts). **(b)** Quantitative analysis of the number (n = 30 cells/condition), size (n = 100 FA/condition), and aspect ratios (n = 100 FA/condition) of focal adhesions based on the immunostaining images. Data are representative of four independent experiments. **p < 0.01 and ****p < 0.0001; two-way ANOVA followed by Tukey's post hoc.

Supporting Information (Figs. S1-S24 &Table S1-S5)

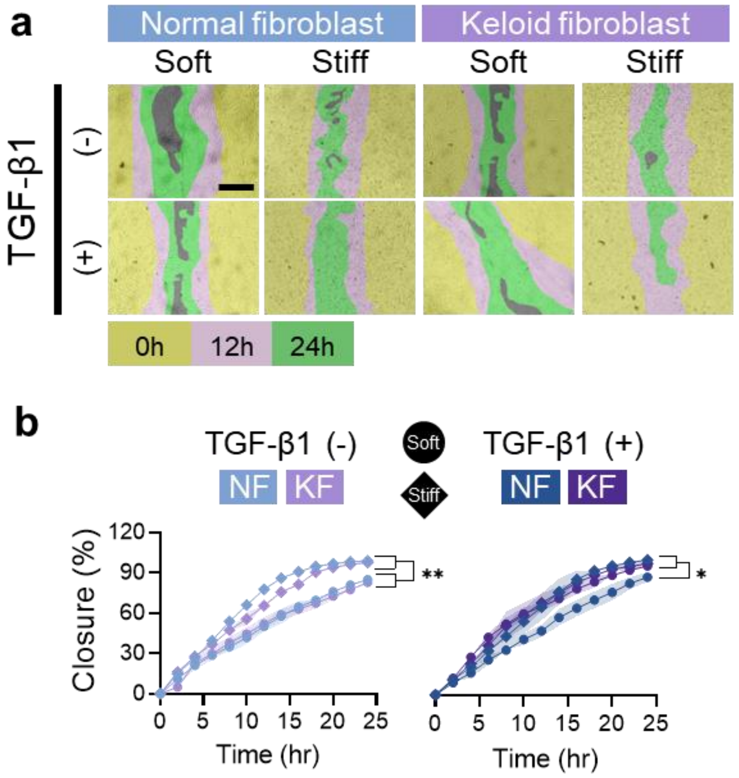

Fig. S20. Scratch-based planar migration assays.

Cells were seeded on either soft or stiff substrates and the migration was monitored with or without TGF-β1 treatment for 24 hours. **(a)** Pseudo-colored cell migration tracking from a representative time-lapse movie and **(b)** quantification (n = 3 replicates from 3 independent experiments). Scale bar, 200 μm. *p < 0.05 and **p < 0.01; two-way ANOVA followed by Tukey's post hoc tests.

Supporting Information (Figs. S1-S24 &Table S1-S5)

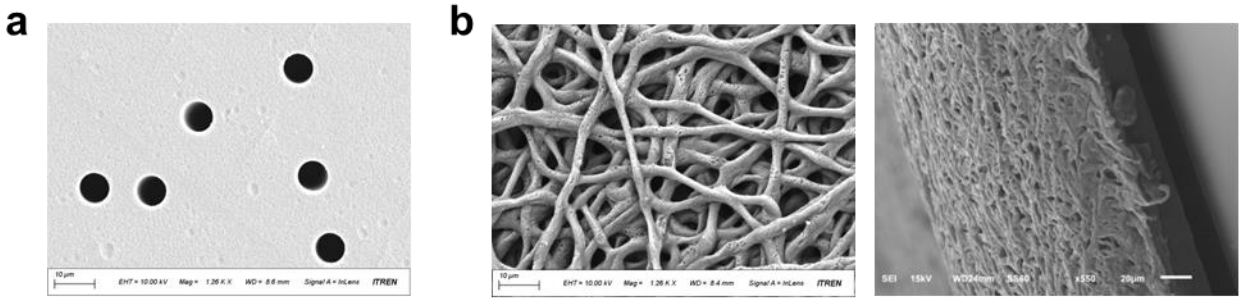

c

Specification	Transwell	PCL fiber
Pore diameter (µm)	8.7± 0.1	3.4 ± 1.1
Fiber diameter (µm)	-	3.1 ± 0.6
Membrane thickness (µm)	10	130.1 ± 2.0

Fig. S21. Morphology and pore size measurement of transwells and PCL fibrous networks used for the confined migration assays.

FE-SEM images of **(a)** transwell and **(b)** PCL fibrous network (left: top view; right: cross-sectional view), along with **(c)** size specifications.

Supporting Information (Figs. S1-S24 &Table S1-S5)

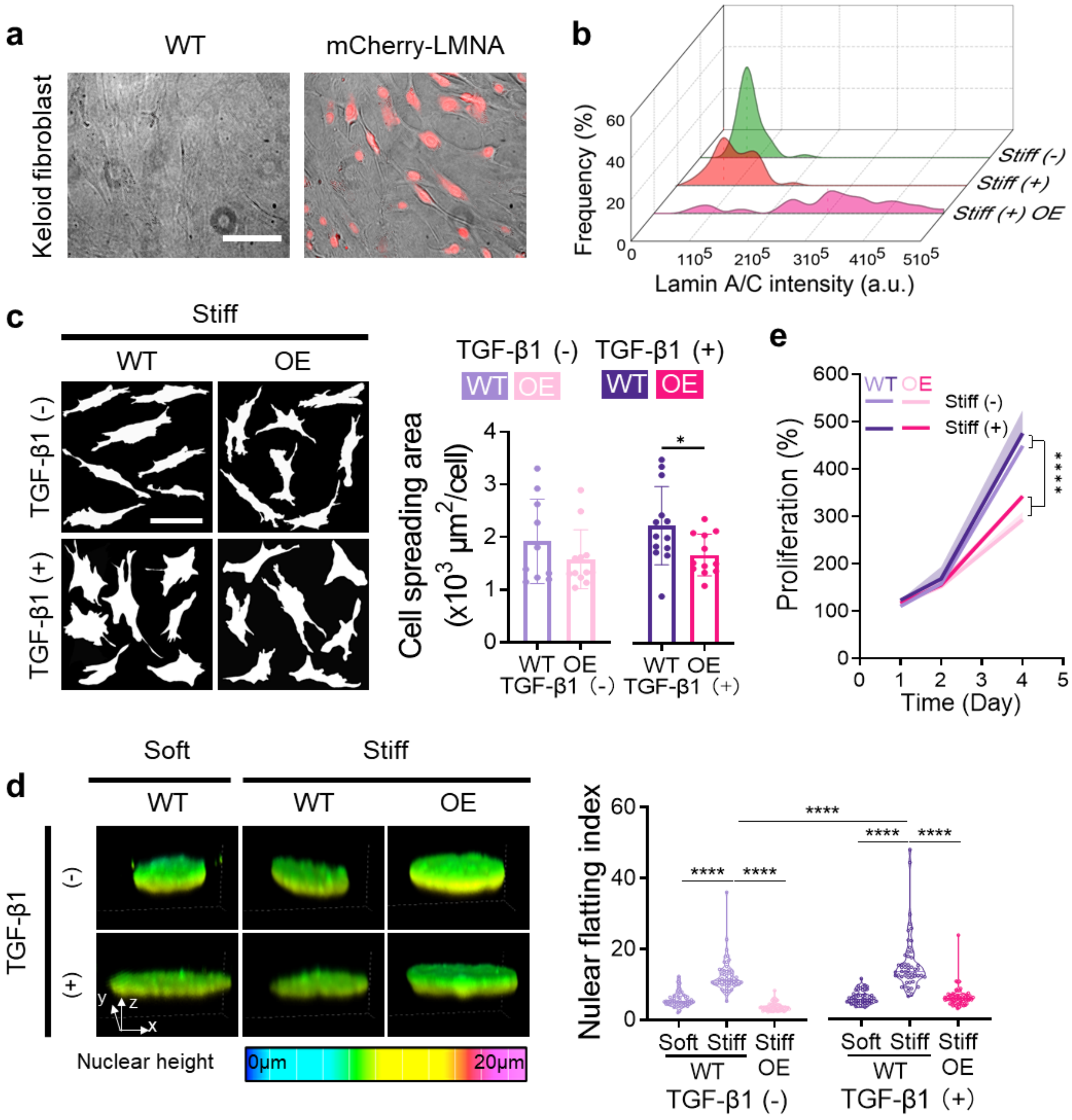

Supporting Information (Figs. S1-S24 &Table S1-S5)

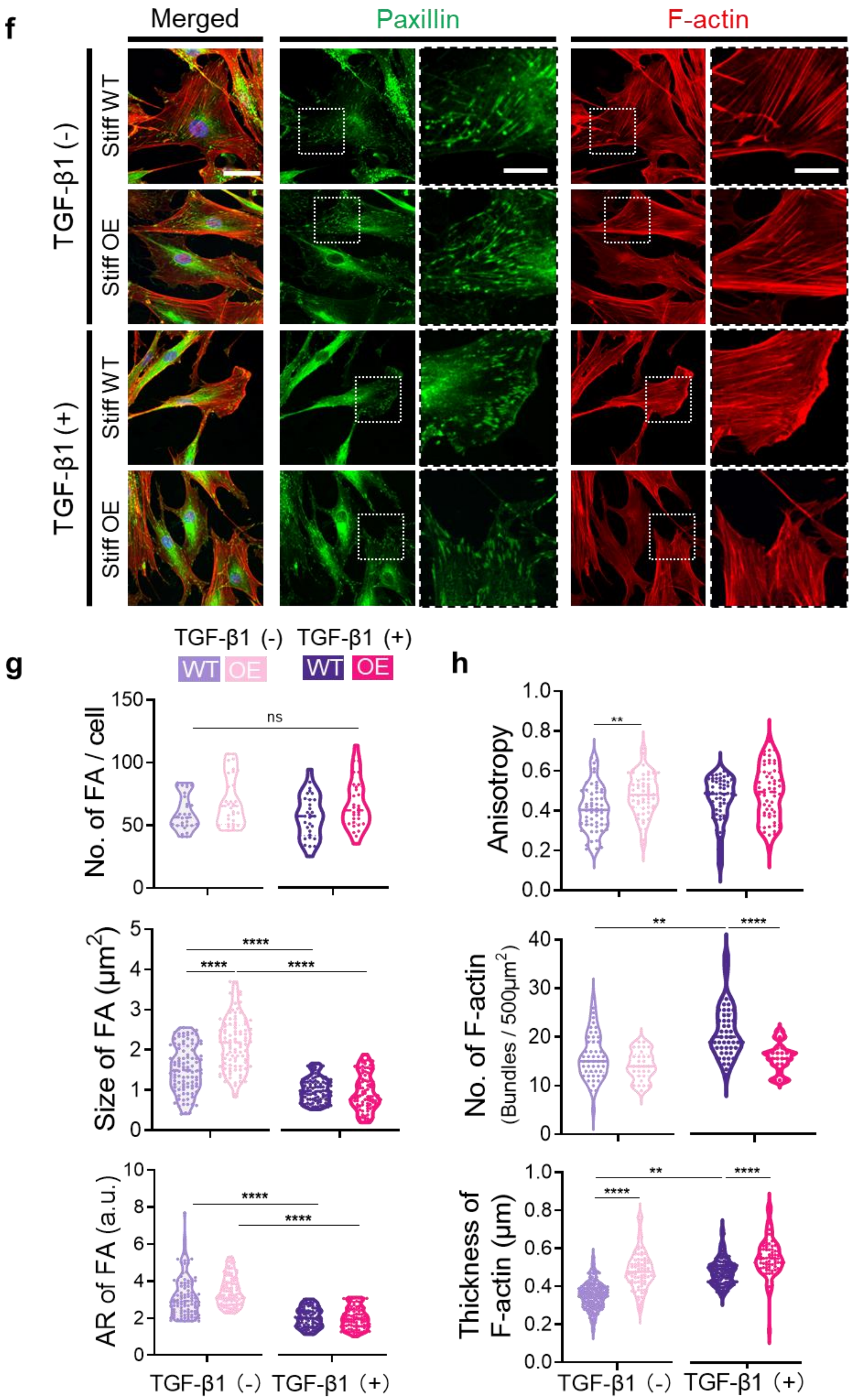

Supporting Information (Figs. S1-S24 &Table S1-S5)

Fig. S22. Characteristics of lamin A/C-overexpressed keloid fibroblasts.

(a) Keloid fibroblasts were transfected with mCherry-LMNA plasmid (red) to overexpress lamin A/C. Wild-type (WT) or lamin A/C-overexpressed (OE) KFs were cultured on stiff substrates for 2 days. **(b)** 3D waterfall plots of lamin A/C intensity and frequency in WT or OE KFs cultured on stiff substrates without or with TGF-β1 (Stiff (-) or Stiff (+), n = 60 cells/condition). **(c)** Representative cell mask images (left) and quantitative analysis of cell spreading area (right, n = 10 - 13 field/condition). **(d)** Representative color-coded images of three-dimensional nuclear morphology (left) along with quantitative analysis of nuclear flatting index (right, n= 53 - 60 cell/condition) in WT and OE KFs cultured on substrates. **(e)** Proliferation rate based on CCK-8 assay (n = 3 replicates/condition). **(f)** Representative co-staining of paxillin (green) and F-actin (red). Scale bar, 50 μm (main images), and 15 μm (inserts). Nuclei are stained with DAPI (blue). **(g)** Quantitative analysis of the number (n = 30 cells/condition), size (n = 100 FA/condition), and aspect ratios (n = 100 FA/condition) of focal adhesions based on the co-staining images. **(h)** Quantitative analysis of the F-actin anisotropy, the number, and thickness of F-actin bundles (n = 30 - 200 cells/condition). Data represent mean ± s.d. of *n* and are representative of four independent experiments. *$p < 0.05$, **$p < 0.01$ and ****$p < 0.0001$; two-way ANOVA followed by Tukey's post hoc tests.

Supporting Information (Figs. S1-S24 &Table S1-S5)

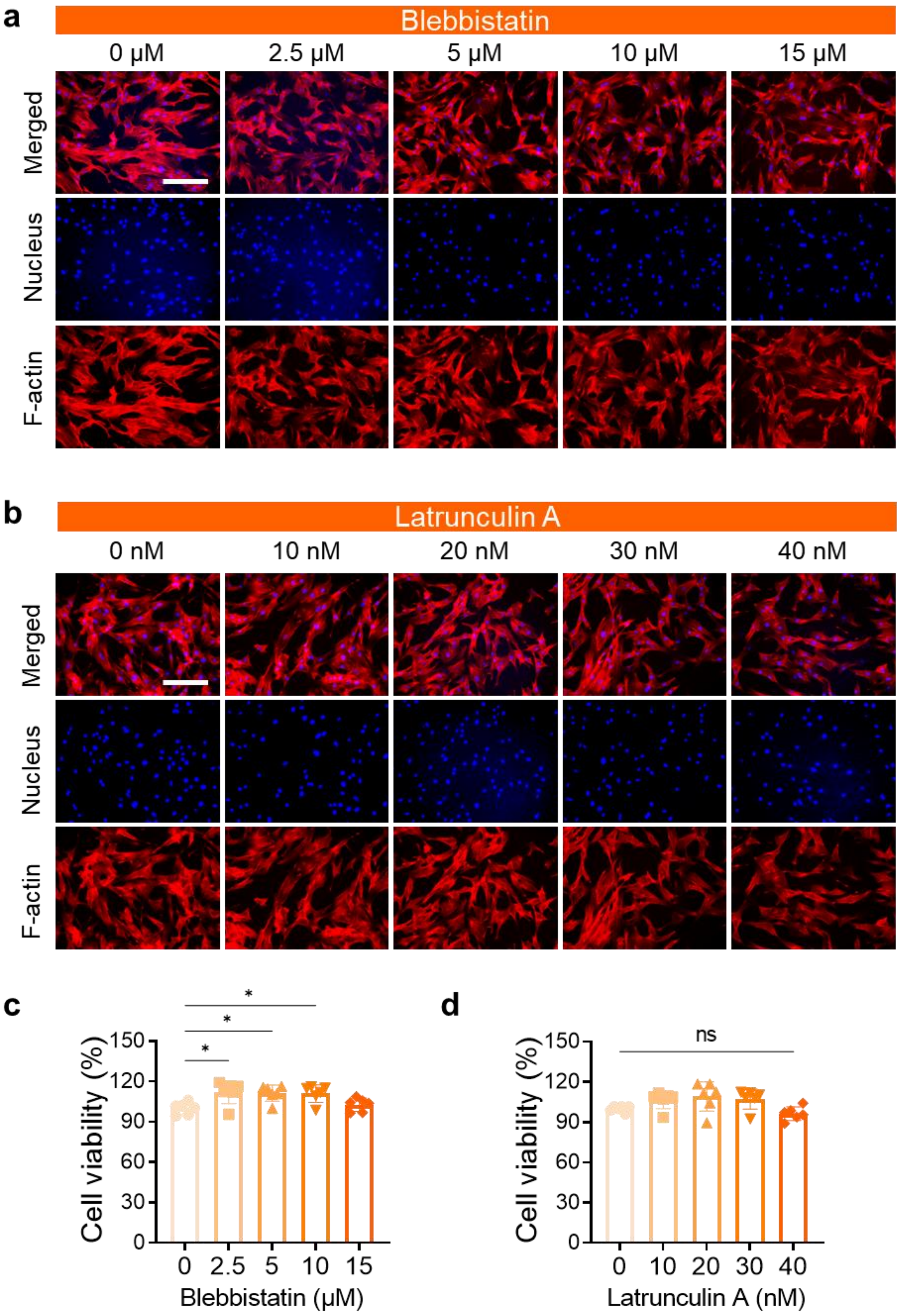

Supporting Information (Figs. S1-S24 &Table S1-S5)

Fig. S23. Dose screening of blebbistatin and latrunculin A on keloid fibroblasts.

(a, b) Representative F-actin staining images of KFs treated with blebbistatin (a) or latrunculin A (b). Scale bar, 200 μm. **(c, d)** Cell viability of KFs treated with blebbistatin (c) or latrunculin A (d) evaluated by CCK-8 assay (n = 6 replicates from 3 independent experiments). *$p < 0.05$; one-way ANOVA followed by Dunnett tests.

Supporting Information (Figs. S1-S24 &Table S1-S5)

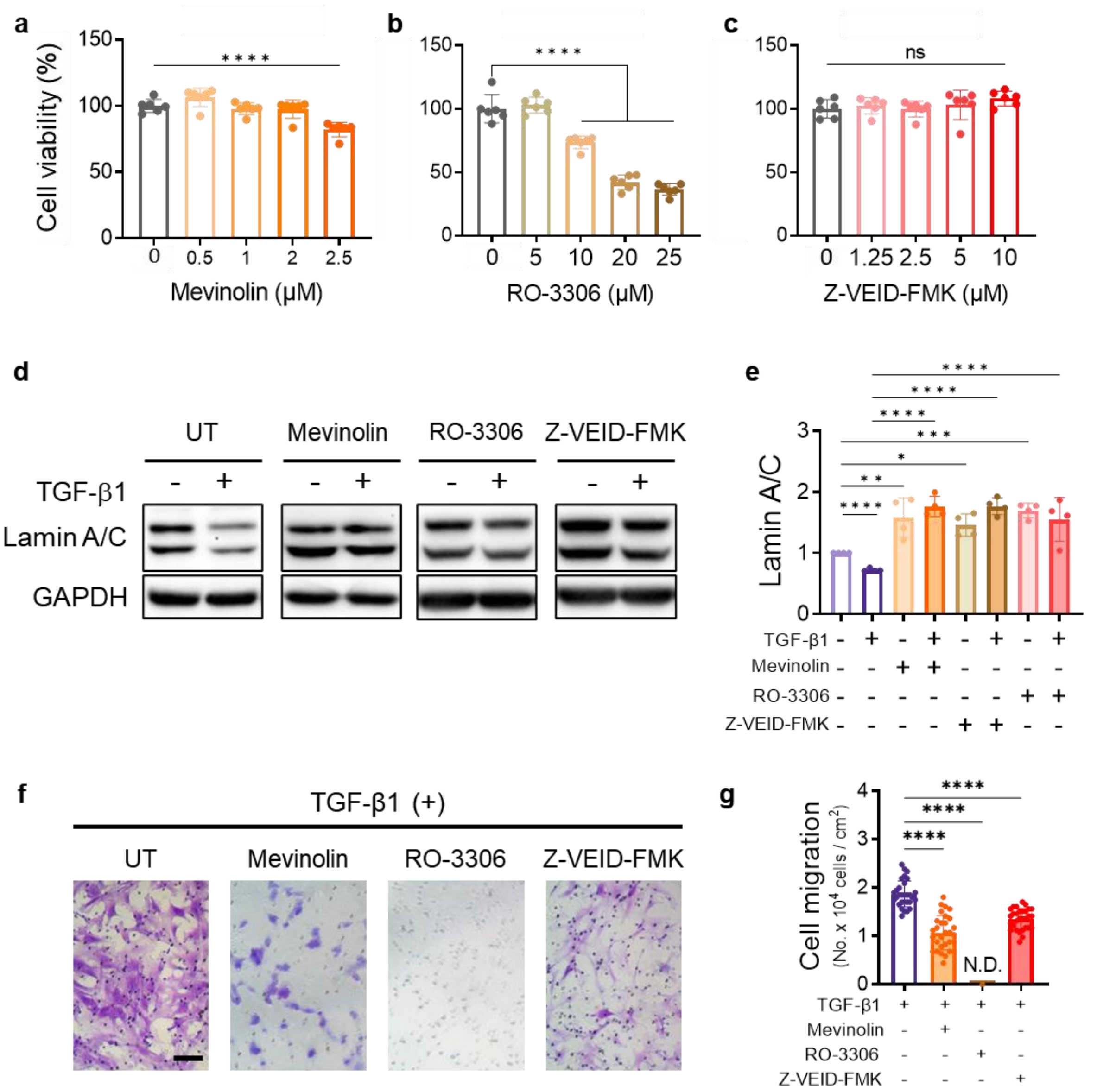

Fig. S24. In vitro treatment of potential pharmaceuticals targeting lamin A/C.

(a-c) Cell viability of KFs treated with Mevinolin (a), RO-3306 (b), or Z-VEID-FMK (c) evaluated by CCK-8 assay (n = 6 replicates from 3 independent experiments). ****p < 0.0001; one-way ANOVA followed by Dunnett tests. **(d)** Representative western blots and (**e**) quantification of lamin A/C in KFs treated with Mevinolin, RO-3306, or Z-VEID-FMK (n = 4 replicates/condition). (**f**) Representative images of crystal violet staining (purple) following the transwell (8 μm-diameter pores) migration assay and (**g**) quantification (n = 30 fields/condition). Scale bar, 100 μm. Data represent mean ± s.d. of n and are representative of at least three independent experiments. *p < 0.05, **p < 0.01, ***p < 0.001 and ****p < 0.0001; two-tailed paired Student's t-test or one-way ANOVA followed by Bonferroni tests.

Supporting Information (Figs. S1-S24 &Table S1-S5)

Table S1. Donor information for human keloid specimens.

Donor	Race	Gender	Age	Specimen	Lesion
**1**	Asian	Female	23 yr	Keloid	Ear
**2**	Asian	Female	30 yr	Keloid	Ear
**3**	Asian	Female	45 yr	Keloid	Ear

Supporting Information (Figs. S1-S24 &Table S1-S5)

Table S2. Antibody used for histology.

Antibody	Company	Cat. No.	Conc.
**α-SMA (D4K9N)**	Cell signaling	19245	1:200
**YAP (D8H1X)**	Cell signaling	14074	1:200
**Lamin A/C**	Abcam	ab108595	1:200

Supporting Information (Figs. S1-S24 &Table S1-S5)

Table S3. Information on dermal fibroblasts.

Cell	Race	Gender	Age	Organ	Cat. No.	Lot No.
**Normal dermal fibroblast**	Caucasian	Female	58 yr	Facial skin	PH10605A	190816-2935
**Keloid fibroblast**	African	Female	35 yr	Keloid skin	CRL-1762	70032510

Supporting Information (Figs. S1-S24 &Table S1-S5)

Table S4. Antibodies used for immunofluorescence staining.

Antibody	Company	Cat. No.	Conc.
**Paxillin**	Abcam	ab32084	1:100
**α-SMA [1A4]**	Abcam	ab7817	1:500
**Smad2/3 (C-8)**	Santa Cruz	sc-133098	1:50
**YAP (63.7)**	Santa Cruz	sc-101199	1:50
**YAP (D8H1X)**	Cell signaling	14074S	1:100
**MTRF-A**	Invitrogen	PA5-99446	1:200
**Lamin A/C**	Abcam	ab108595	1:200
**Anti-acetyl-Histone H3**	Sigma	06-599	1:100
**HDAC3 (D2O1K)**	Cell signaling	85057	1:100
**Di/Tri-Methyl-Histone H3 (Lys9) (6F12)**	Cell signaling	5327	1:100
**Tri-Methyl-Histone H3 (Lys27) (C36B11)**	Cell signaling	9733	1:100
**Tri-methyl H3K4me**	Abcam	ab8580	1:100
**Fluorescein (FITC) AffiniPure Donkey Anti-Mouse IgG (H+L)**	Jackson ImmunoResearch	715-095-150	1:200
**Fluorescein (FITC) AffiniPure Donkey Anti-Rabbit IgG (H+L)**	Jackson ImmunoResearch	711-095-152	1:200

Supporting Information (Figs. S1-S24 &Table S1-S5)

Table S5. Primers used for qRT-PCR.

Gene	Sequence
**hGAPDH**	F: 5'-GCACCGTCAAGGCTGAGAAC-3'<br>R: 5'-CCACTTGATTTTGGAGGGATCT-3'
**hα-SMA**	F: 5'-CTGTTCCAGCCATCCTTCAT-3'<br>R: 5'-TCATGATGCTGTTGTAGGTGGT-3'
**hHDAC1**	F: 5'-AGTGCGGTCGTCTTACAG-3'<br>R: 5'-CCTCCCAGCATCAACATA-3'
**HDAC2**	F: 5'-GGAACAGGAGACTTGAGGGAT-3'<br>R: 5'-CAGCACCACATTGTAACACGAC-3'
**hHDAC3**	F: 5'-GCTGCTGGACGGATGAGA-3'<br>R: 5'-CTGGATGGAGCGTGAAGT-3'
**hHDAC8**	F: 5'-CCACCTTCCACACTGATGCT-3'<br>R: 5'-GCTGGGCAGTCATAACCTAGC-3'
**hHDAC4**	F: 5'-TCAAGGCACCCGAGAAGA-3'<br>R: 5'-ACGACGGAGACAAACAGACAAG-3'
**hHDAC5**	F: 5'-AACTCTGTAGCCATCACAACCA-3'<br>R: 5'-CCCTCCGCCAACCACTT-3'
**hHDAC7**	F: 5'-TCTCGTGAGCTAAAGAATGG-3'<br>R: 5'-CTGTTGAATGATCTGCATGG-3'
**hHDAC9**	F: 5'-AGCCCATCTCGCCTTTA-3'<br>R: 5'-TTGCTGCGGTTGCTGAAT-3'
**hHDAC6**	F: 5'-CCGCCCGAAGTGTAAAT-3'<br>R: 5'-AGACCTGCCAGTCATCCC-3'
**hHDAC10**	F: 5'-GCCGTCTACTTCCACCCG-3'<br>R: 5'-GCACAACTCCCGCCATC-3'
**hSIRT1**	F: 5'-CTTCAGTTGCCGAAACAGTAAGAA-3'<br>R: 5'-CATCAAGCCGTTTACTAATCTGC-3'
**hSIRT2**	F: 5'-CTTCGCCCTCGCCAAGGAACTCTA-3'<br>R: 5'-CCGCCACTCGCTCCAGGGTGTCTA-3'
**hSIRT6**	F: 5'-AGGGACAAACTGGCAGAGC-3'<br>R: 5'-TTAGCCACGGTGCAGAGC-3'
**hSIRT7**	F: 5'-GCAGAGCAGACACCATCC-3'<br>R: 5'-GTTCACGATGTAAAGCTTCG-3'

Supporting Information (Figs. S1-S24 &Table S1-S5)

**hHDAC11**	F: 5'-CGGAAAATGGGGCAAAGTGA-3'<br>R: 5'-CAACAGCAAAGGACCACTTG-3'
**hHAT1**	F: 5'-GCTCCCACTTGGATCTCGAC-3'<br>R: 5'-GCACCAAATCCCTAAAAGAGAAGG-3'
**hEzh2**	F: 5'-CATTTCATACGCTCTTCTGTCGAC-3'<br>R: 5'-CCCTCCAGATGCTGGTAACACT-3'
**hSuv39H1**	F: 5'-ATCCGCGAACAGGAATATTACC-3'<br>R: 5'-GAGGATACGCACACACTTGAGATT-3'
**hSuv39H2**	F: 5'-CCCAAATCTTCAGGTGTTCAATG-3'<br>R: 5'-GGTTCTTGTGGAAAACAATGCTATT-3'
**hTwist1**	F: 5'-TAGAAGTCTGAACACTCGTT-3'<br>R: 5'-AATTCCTCTGATTGTTACCATT-3'
**hG9a**	F: 5'-CATTTCCGCATGAGTGATGATGT-3'<br>R: 5'-GGCAGAACCTAACTCCTCCGA-3'
**hSETDB1**	F: 5'-CCCAGGATCTGCATAAAGGA-3'<br>R: 5'-TCAGCAGGAGGGTGGTAATC-3'
**hMLL1**	F: 5'-GAAGTGGTTCCTGAGAATGG-3'<br>R: 5'-CACAGTCGGAGAGATCATTTAG-3'
**hMLL2**	F: 5'-TGATCGAGAAAGTGCAAGAG-3'<br>R: 5'-CTGGTGGTAACGGAACTTATAG-3'
**hMLL3**	F: 5'-AGTCTTCAGGAGGGTCTATG-3'<br>R: 5'-CACAGGGAAGAGTGCTTTAG-3'
**hMLL4**	F: 5'-CTCTGGATGGGATTGATGCT-3'<br>R: 5'-CGTGGCTCTTCCTGTTCTTC-3'
**hSET1A**	F: 5'-CATCGAATACGTGGGTCAGA-3'<br>R: 5'-AATGCCCTCCTGCACGTA-3'
